# Machine Learning‐Enhanced Analysis of Exosomal Surface Sialic Acid Using Surface‐Enhanced Raman Spectroscopy for Ovarian Cancer Diagnosis and Therapeutic Monitoring

**DOI:** 10.1002/advs.202518190

**Published:** 2026-01-21

**Authors:** Lili Cong, Jiaqi Wang, Sijun Huang, Xiaxia Man, Yi Guo, Shuping Xu, Songling Zhang

**Affiliations:** ^1^ Department of Gynecological Oncology, Gynecology and Obstetrics Center The First Hospital of Jilin University Changchun P. R. China; ^2^ State Key Laboratory of Supramolecular Structure and Materials, College of Chemistry Jilin University Changchun P. R. China; ^3^ Jilin Provincial Key Laboratory of Women's Reproductive Health Changchun P. R. China; ^4^ Key Laboratory for Molecular Enzymology and Engineering Ministry of Education School of Life Sciences Jilin University Changchun P. R. China

**Keywords:** exosomal sialic acid, machine learning algorithms, ovarian cancer diagnosis, SERS nanosensor, therapeutic monitoring

## Abstract

Currently, the absence of ovarian cancer (OC)‐specific biomarkers impedes the development of precise noninvasive diagnostic and monitoring strategies. Exosomal surface sialic acid (SA), a key mediator of intercellular communication and disease progression, emerges as a promising biomarker, though its role in OC remains unclear. Conventional exosome isolation and detection methods exhibit limited clinical utility. Herein, we developed a CD63 aptamer‐functionalized gold array chip integrated with a surface‐enhanced Raman scattering (SERS) nanosensor for sensitive SA analysis. The chip efficiently isolated exosomes from clinical serum, while the nanosensor selectively bound exosomal SA via molecular recognition, thereby altering the SERS intensity ratio of the nanosensor. More importantly, machine learning can discern SA signatures from SERS spectra, achieving 93% accuracy in OC diagnosis. The longitudinal monitoring of SA throughout the entire treatment period (preoperative, postoperative, and chemotherapy) revealed a potential correlation with treatment response as indicated by clinical markers (CA125, HE4), demonstrating the utility of exosomal SA in precision treatment evaluation. This provides a powerful tool for the diagnosis and treatment monitoring of OC and plays a critical role in precision medicine.

## Graphical abstract

Machine learning‐assisted surface‐enhanced Raman spectroscopy analysis of exosomal sialic acid for ovarian cancer diagnosis, as well as independent monitoring of exosomal sialic acid expression levels across different treatment periods, reveals a potential correlation with treatment response.

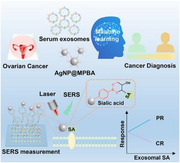

## 1 Introduction

Ovarian cancer (OC) is a predominant malignancy within the female reproductive system, exhibiting the highest mortality rate among gynecological cancers [1]. Currently, tissue biopsy remains the most prevalent diagnostic approach [2]. Nevertheless, the finite number of tissue samples obtained often fails to capture the tumor's inherent heterogeneity and is incapable of providing dynamic monitoring of tumor progression. Moreover, this invasive procedure may inadvertently increase the risk of metastasis, thereby resulting in diminished survival rates and unfavorable prognoses [3]. In contrast, liquid biopsy technology, which involves the detection and analysis of bodily fluids (e.g., blood, urine), offers a minimally invasive alternative with high sensitivity and specificity, presenting a promising avenue for early cancer detection [4]. However, the successful implementation of this technology hinges on the identification of appropriate, specific biomarkers and the selection of highly sensitive detection methodologies.

Exosomes are nanoscale extracellular vesicles (30–150 nm) secreted by parent cells into the extracellular matrix (ECM), carrying molecular characteristics that reflect their cells of origin [5, 6]. They are actively released by most cells and circulate stably in body fluids [7, 8]. Tumor‐derived exosomes possess characteristic nucleic acids, proteins, lipids, and glycans, and are abundantly present in bodily fluids, making them promising biomarkers for non‐invasive cancer detection via liquid biopsy [9, 10, 11]. In OC studies, although the deep pelvic location of the ovaries, these tumor‐associated exosomes exert systemic effects by aiding in the establishment of pre‐metastatic niches in lymph nodes and organ‐specific distant sites [12].

Concerning glycosylation, lectin array technology revealed that exosomes contain glycan features characteristic of the parent cell membrane [13, 14]. Sialic acid (SA) is an important and unique form of abnormal glycosylation. Its residues can be linked to the basal sugar via α2,3‐, α2,6‐, or α2,8‐glycosidic bonds, meaning that the C2 position of SA forms α‐glycosidic bonds with the C3, C6, or C8 positions of another sugar molecule, respectively [15]. This diverse mode of attachment typically positions SA at the terminal end of *N*‐linked and *O*‐linked glycan chains on the exosome surface, thereby forming the structurally rich sialylated glycans [16, 17]. Mechanistically, OC cells overexpress ST6GAL1 (sialyltransferase), which leads to increased levels of α2, 3‐linked SA on the exosome surface [17, 18]. This modification facilitates the binding of exosomes to sialic acid‐binding immunoglobulin‐like lectin (Siglec) receptors on immune cells, forming an immunosuppressive microenvironment and promoting tumor progression [19]. Additionally, the negative charge of SA leads to enhanced cell‐cell adhesion, actin contraction, and migration [20]. Substantial evidence demonstrates a significant correlation between elevated SA levels and the incidence of OC [21, 22, 23, 24]. Studies have also revealed the functional impact of glycan alterations in OC, indicating that sialylation serves as a key regulator of OC progression, metastasis, and chemoresistance, highlighting its potential as a therapeutic target [25]. Collectively, these findings suggest that exosome‐associated SA may serve as ideal targets for non‐invasive early diagnosis of OC.

However, detecting exosomes in complex clinical samples, such as serum, faces two primary challenges. First, efficient isolation of exosomes remains technically demanding. Current methods, including ultracentrifugation, density gradient centrifugation, and ultrafiltration [26], are limited by large sample requirements, high equipment costs, and labor‐intensive procedures. Second, the structural complexity of exosomal glycans complicates direct analysis of sialic acid modifications. Emerging approaches, such as lectin‐based recognition, metabolic glycan labeling, chemical modification, and enzyme‐assisted strategies [27, 28, 29, 30], offer promising alternatives but are constrained by the need for exosome labeling, potential structural/functional alterations, and sensitivity issues Furthermore, glycan analysis demands more sophisticated instrumentation (e.g., high‐end mass spectrometry), specialized bioinformatics tools, and comprehensive databases compared to nucleic acid or protein analysis [31, 32]. Although microfluidic platforms show potential for clinical EV detection by translating glycan signatures into magnetic signals [33], their fabrication requires advanced equipment and technical expertise.

Surface‐enhanced Raman scattering (SERS) is a powerful vibrational spectroscopy technique that enhances molecular signal intensity through localized surface plasmon resonance [34, 35], providing ultra‐high sensitivity for molecular characterization and demonstrating significant potential in bio‐detection and therapeutic applications. Recent advancements have highlighted the efficacy of both label‐free and label SERS strategies in exosome‐based cancer diagnosis and treatment monitoring [36, 37, 38]. Nevertheless, the clinical translation of these approaches faces substantial challenges, primarily due to the compositional complexity of biological samples and the weakness of characteristic peaks that are difficult to distinguish using traditional analytical methods. The integration of machine learning algorithms serves as a transformative solution that overcomes the technical limitations of traditional SERS analysis by enabling automated feature extraction, nonlinear modeling, and efficient data processing. This approach enhances the accuracy and efficiency of analyzing complex samples, thereby significantly advancing SERS‐based clinical applications [39, 40].

**SCHEME 1:**
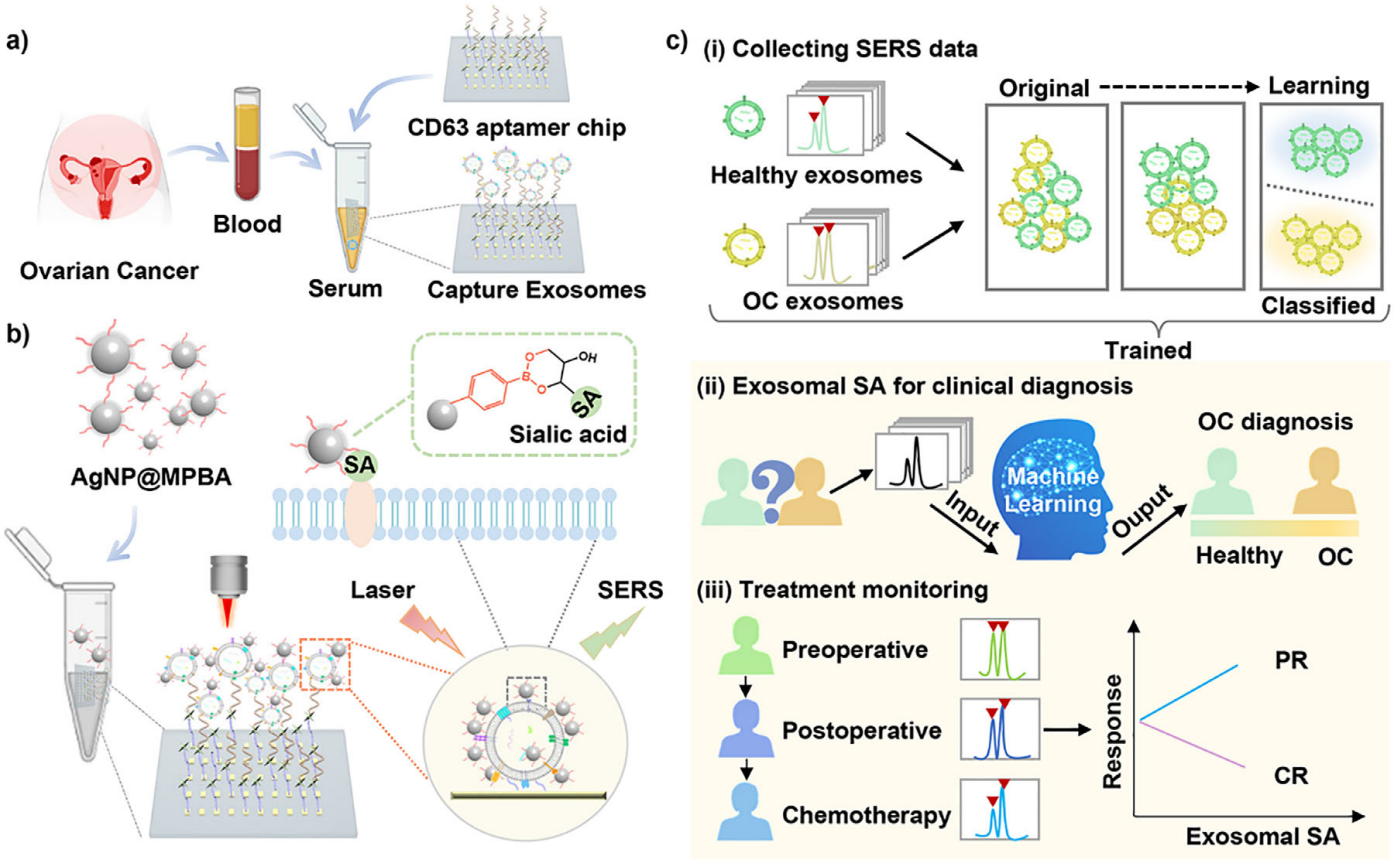
(a) Schematic of clinical sample processing: Serum‐derived exosomes from OC patients were captured via a silicon‐based gold array‐CD63 aptamer chip. (b) Operational principle of the SERS nanosensor for detecting exosomal SA. (c) SERS data from serum‐derived exosomal SA in healthy individuals and OC patients. The machine learning algorithm was implemented to (i) classify exosomes, (ii) diagnose OC. (iii) Dynamically monitor exosomal SA levels across therapeutic periods (preoperative, postoperative, and chemotherapy).

In summary, to advance exosome‐based clinical diagnostics, we utilized exosomal SAs as a diagnostic biomarker for OC and integrated SERS spectroscopy with machine learning algorithms to improve OC diagnosis. Our approach includes three key steps: (1) A gold array‐based exosome capture chip functionalized with CD63 aptamers, enabling microliter‐scale sample processing and rapid exosome enrichment (Scheme 1a). (2) Under physiological pH conditions, 4‐mercaptophenylboronic acid (MPBA) can covalently bind to the *cis*‐diol moiety of SA to form reversible five‐ or six‐membered cyclic boronates. Therefore, we designed AgNP@MPBA nanoprobes, which function as both a Raman reporter and an exosome‐specific SA recognition element, and were able to assess SA expression levels by changes in the SERS peak intensity ratio of MPBA (Scheme 1b). (3) Comparing multiple machine learning algorithms to analyze exosomal SA signatures, thereby establishing a more robust diagnostic model for distinguishing healthy individuals from OC patients (Scheme 1ci,ii). Furthermore, we monitored dynamic changes in exosomal SA levels across different treatment phases (preoperative, postoperative, and chemotherapy). We correlated these changes with treatment response as indicated by the clinical markers CA125 and HE4 (Scheme 1ciii). This integrated platform not only provides a potential tool for OC diagnosis but also offers insights into personalized treatment monitoring through exosomal SA profiling.

## 2 Results

### 2.1 Morphological Characterization of Capture Chips and Exosomes

**FIGURE 1:**
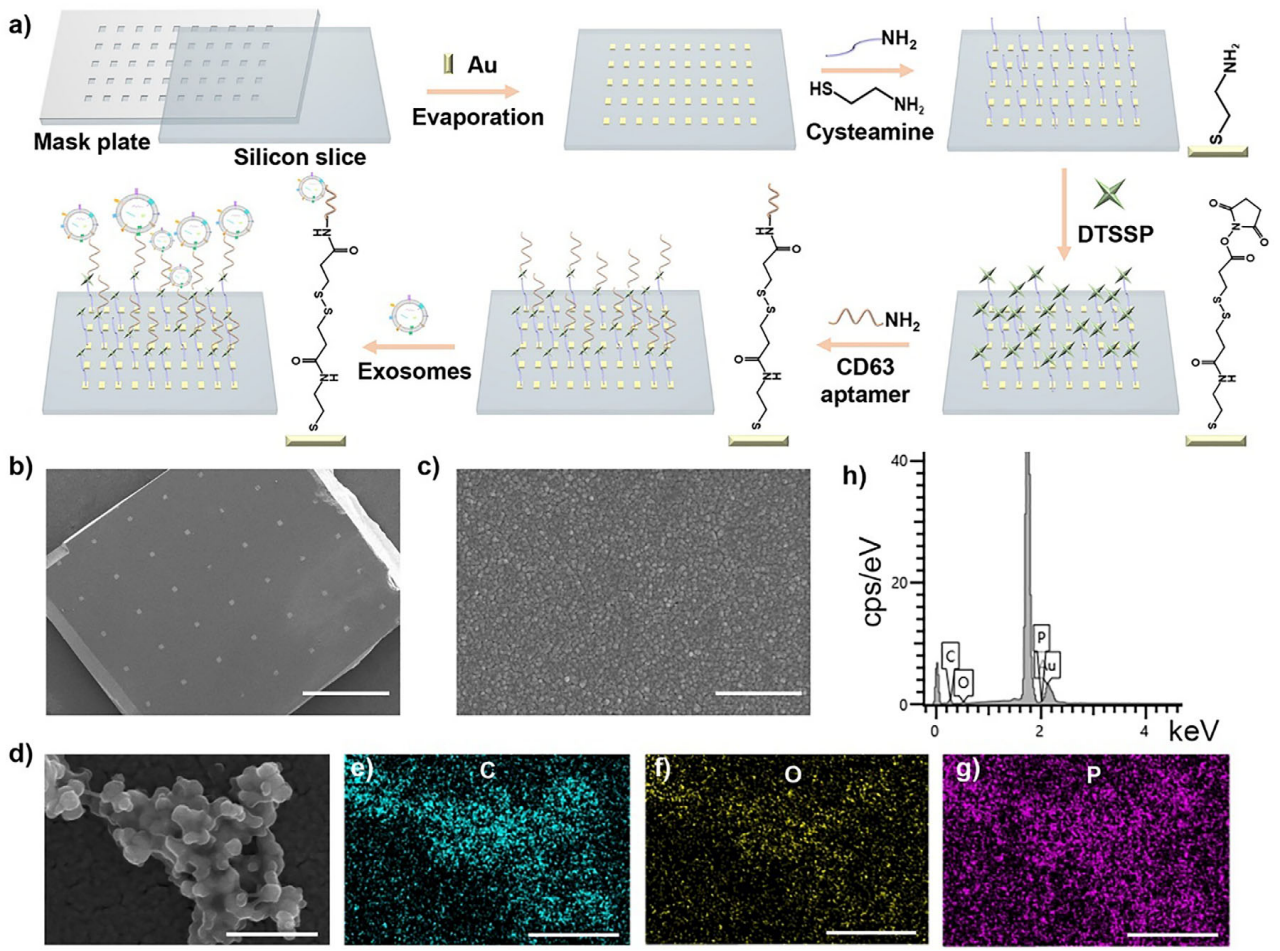
(a) Schematic illustration of silicon‐based gold array film fabrication for exosome capture. (b) SEM image of the exosome capture chip with silicon‐based gold array films. Scale bar is 1 mm. (c) Magnified SEM image of the gold array in (b). Scale bar is 500 nm. (d) SEM image of serum‐derived exosomes captured on the chip, along with corresponding elemental mapping images of C (e), O (f), and P (g). Scale bar is 500 nm. (h) Energy spectrum of exosomes when they were captured by the chip.

Current exosome enrichment methodologies employ membrane protein‐targeting antibodies and CD63 aptamers [41, 42] and interfacial interactions such as static interaction, molecular imprinting, rare‐earth chelation, and reversible zwitterionic coordination [37, 43, 44, 45, 46]. We engineered a silicon‐gold array chip functionalized with CD63 aptamers for serum exosome isolation (Figure 1a), integrating exosome capture with SERS localization capabilities. The gold array film fabricated via evaporation on silicon wafers (Figure 1b) exhibits uniform unit dimensions (60 µm × 60 µm) with a height of 13–15 nm (Figure S2a–d). Surface roughness analysis revealed roughness average (Ra) values of 0.27 nm (R1‐R2 directions) and 0.25 nm (R3‐R4 directions) (Figure S2b,e,f), demonstrating uniform deposition. SEM imaging further confirmed the gold film's high compactness and morphological homogeneity (Figure 1c).

Next, the specific binding capacity of the CD63 aptamer toward exosomes was evaluated by SEM imaging. Chips assembled with or without the CD63 aptamer were co‐incubated with OC patients' serums. As shown in Figure S3, chips without assembled CD63 aptamers failed to capture exosomes, indicating no non‐specific adsorption of serum‐derived exosomes. In contrast, chips assembled with CD63 aptamers effectively enriched exosomes after incubation with serum (Figure 1d). Elemental mapping analysis (Figure 1e–h) revealed the primary components of exosomes, including carbon elements in proteins and lipids, oxygen elements in proteins and polysaccharides, and characteristic phosphorus distribution in phospholipid bilayers. These elementals provide conclusive evidence for the successful isolation of exosomes with preserved structural integrity. Furthermore, fluorescence confocal microscopy revealed the presence of CD9 and CD63 proteins on the chip surface (Figure S4), further validating its ability to enrich exosomes. The above findings collectively demonstrate that only chips assembled with CD63 aptamers can enrich exosomes, indicating that CD63 aptamers possess the ability to specifically recognize exosomes.

Subsequently, we compared the chip‐capture method with the conventional method (e.g., ultracentrifugation) for exosome isolation. As shown in Figure S5, serum‐derived exosomes isolated by ultracentrifugation contain co‐precipitated lipoproteins or large vesicles, requiring subsequent purification to obtain pure exosomes. Additionally, high centrifugal forces can rupture vesicle membranes, compromising exosome integrity. In contrast, our chip identifies the positive protein CD63 on exosomal surfaces via the CD63 aptamer. This protein is unique to exosomes, and we can distinguish exosomes from lipoproteins and large vesicles according to their overexpressed CD63. Thus, we can enrich exosomes from serum via our designed chip without additional purification, beyond the ultracentrifugation method.

### 2.2 Theoretical Simulations and Experiments Reveal MPBA in Response to SA

**FIGURE 2:**
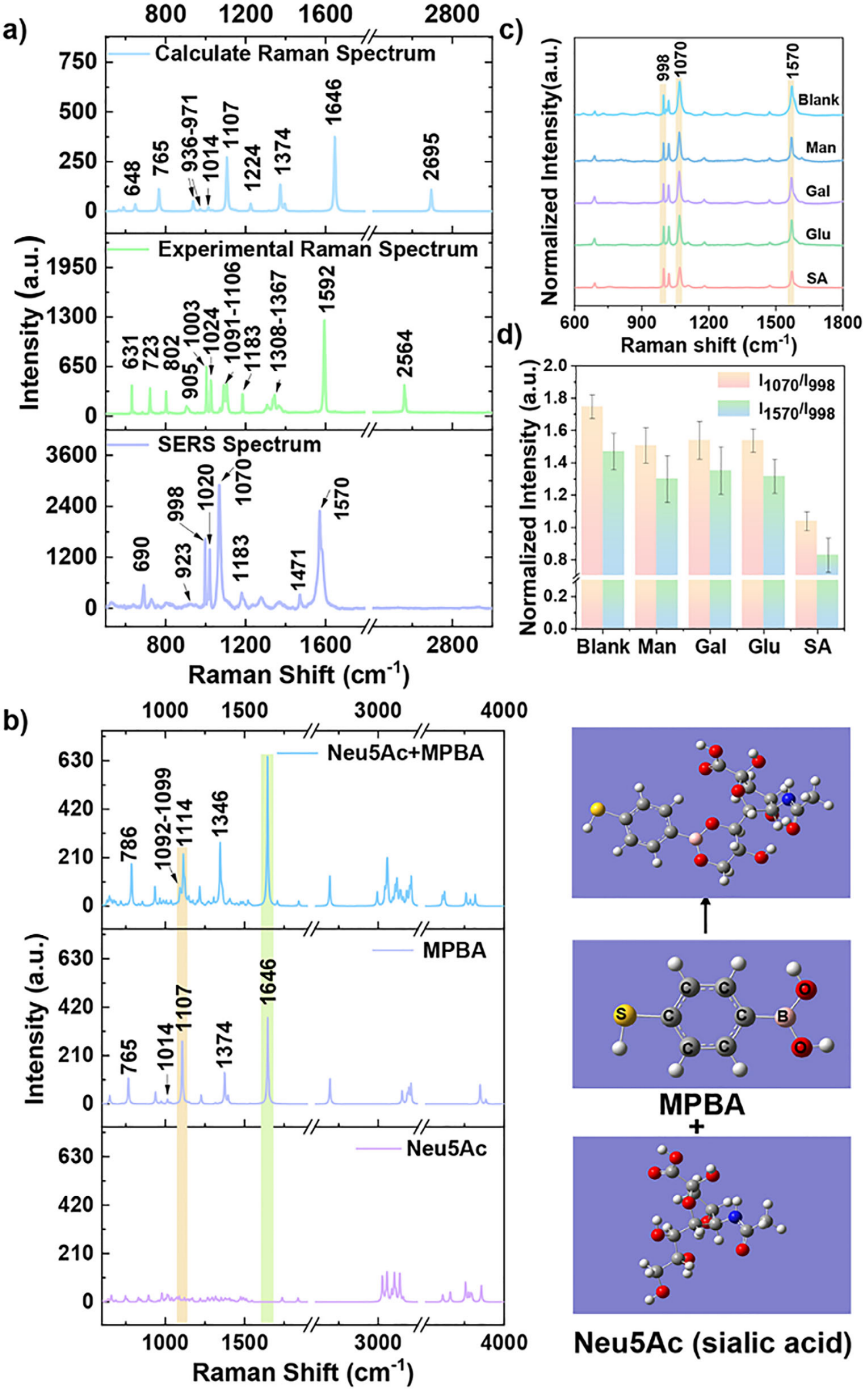
(a) DFT calculated (top) and experimental (middle) Raman spectra of MPBA molecules and SERS spectra of AgNP@MPBA nanosensors (bottom). The experimental Raman spectrum was obtained by measuring solid MPBA powder. λ_ex_ = 632.8 nm, t = 10 s, accumulation = 1 time. The SERS spectrum was acquired by measuring AgNP@MPBA in aqueous solution. λ_ex_ = 632.8 nm, t = 2 s, accumulation = 1 time. (b) DFT calculation of the Raman spectra for MPBA‐bound SA (top), MPBA (middle), and SA (Neu5Ac, bottom). (c) Mean SERS spectra of AgNP@MPBA after 1 h incubation with 1.0 mM of mannose, galactose, glucose, and SA, respectively. The mean spectrum is the average of three spectra. (d) The histograms at I_1070_/I_998 cm‐1_ and I_1570_/I_998 cm‐1_ for the blank, mannose, galactose, glucose, and SA groups, respectively. Error bars represent the standard deviation.

To explore the molecular mechanism through which SA (Neu5Ac) affects MPBA vibrational modes, the Raman spectrum of MPBA was calculated using density functional theory (DFT). This enabled the interpretation of experimentally recorded Raman and SERS bands, as illustrated in Figure 2a and Table S1. To elucidate the MPBA‐Neu5Ac interaction, we analyzed the changes in Raman spectra before and after molecular binding. Since the head‐isomer position of SA in sialoglycans is linked to the glycan via a glycosidic bond, DFT simulations were conducted using the binding of 4‐MPBA to the C7‐C9 position of SAs as a model [47, 48, 49]. Following Neu5Ac (SA) binding, four characteristic peaks (765, 1107, 1374, and 1646 cm^−1^) in the calculated MPBA Raman spectrum exhibit significant shifts (Figure 2b). Specifically, the γ_CH_+γ_CCC_ (765 cm^−1^) and ν_BO_ (1374 cm^−1^) vibrational modes exhibit Raman shifts. The β_CCC_+ν_CS_+β_OH_ mode shifts from 1107 cm^−1^ to 1092–1099 cm^−1^ with reduced intensity. Conversely, the ν_CC_ mode at 1646 cm^−1^ shows an enhanced trend. Figure 2a displays the SERS spectrum of the AgNP@MPBA nanosensors in aqueous solution, which differs significantly from the Raman spectrum. This is primarily attributed to the selectivity rules governing the SERS enhancement mechanism [50]. In contrast, the 765 and 1374 cm^−1^ modes in the computational spectrum lack corresponding SERS signals, while 1107 and 1646 cm^−1^ respond to 1070 and 1570 cm^−1^ in the SERS spectrum. Additionally, the β_CCC_+β_CH_ mode exhibits minimal intensity at 1014 cm^−1^, corresponding to peaks at 998 and 1020 cm^−1^ in the SERS spectrum. Since the characteristic peak at 998 cm^−1^ in the SERS spectrum of MPBA remained unchanged before and after SA binding [51], all SERS spectra were normalized at 998 cm^−1^. Based on these calculations, the peaks at 1070 and 1570 cm^−1^ in the SERS spectrum may be influenced by SA interactions. This is attributed to the disruption of the C_2v_ structural symmetry of 4‐MPBA upon its interaction with glycans (SAs), leading to changes in the C─C stretching vibration (1570 cm^−1^) of the phenyl group and the B─OH stretching (1070 cm^−1^) [52]. The peak at 1070 cm^−1^ represents the B─OH stretching vibration, which undergoes the most significant change during conversion into cyclic borate due to interaction with glycans (SAs) [53, 54]. To verify whether experimental results align with simulation results, we first confirmed the successful preparation of AgNP@MPBA (Figure S6a–d) and demonstrated its excellent stability (Figure S6e,f). Further exploration of MPBA binding to SAs revealed significant intensity changes at 1070 and 1570 cm^−1^ (Figure 2c). Specifically, the intensity at 1070 cm^−1^ decreases experimentally, consistent with calculation results. However, the experimental peak intensity at 1570 cm^−1^ decreases, contrary to the calculated results. Referring to previous literature, the intensities of both the phenyl C─C stretching (1570 cm^−1^) and B─OH stretching (1070 cm^−1^) vibrations decrease [51, 53, 54]. We believe our experimental findings are reasonable under our experimental conditions, but require further investigation. With this in mind, this study evaluates the expression of exosomal SA primarily by monitoring changes in the B─OH stretching signal (1070 cm^−1^), with the C─C stretching signal 1570 cm^−1^) serving as an auxiliary indicator.

The selectivity of AgNP@MPBA toward SA was also verified. Under physiological conditions at pH 7.4, the AgNP@MPBA was incubated with other monosaccharide molecules (e.g., mannose, galactose, and glucose) and compared with SA. Figure 2c displays the SERS spectral changes upon binding of the AgNP@MPBA with four monosaccharide molecules, respectively. Further comparisons were performed on the peak intensity ratios at I_1070_/I_998 cm‐1_ and I_1570_/I_998 cm‐1_ (Figure 2d). The results revealed that AgNP@MPBA exhibited the most pronounced reduction in these ratios upon interaction with SA, compared with other monosaccharides, demonstrating its higher specificity for SA. This observation is consistent with previous results, which indicates that the binding constant of MPBA with SA is significantly higher than that with other monosaccharides [55].

Next, the sensing performance of AgNP@MPBA nanoprobes for SA was further evaluated. As shown in Figure S7, the intensity of the B‐OH peak at 1070 cm^−1^ decreased with increasing SA concentration. A calibration curve was constructed using the standard addition method and plotted. The peak intensity of B‐OH (1070 cm^−1^) varied with different SA concentrations (5.0 × 10^−7^ mol/L to 5.0 × 10^−4^ mol/L). The SERS response exhibited linearity in the range 1.0 × 10^−6^ to 5.0 × 10^−4^ mol/L. The limit of detection (LOD) and limit of quantification (LOQ) were determined to be 1.0 × 10^−6^ mol/L, demonstrating acceptable detection sensitivity.

### 2.3 Analysis of SA Expression in Exosomes Derived From Healthy Individuals and Ovarian Cancers

**FIGURE 3:**
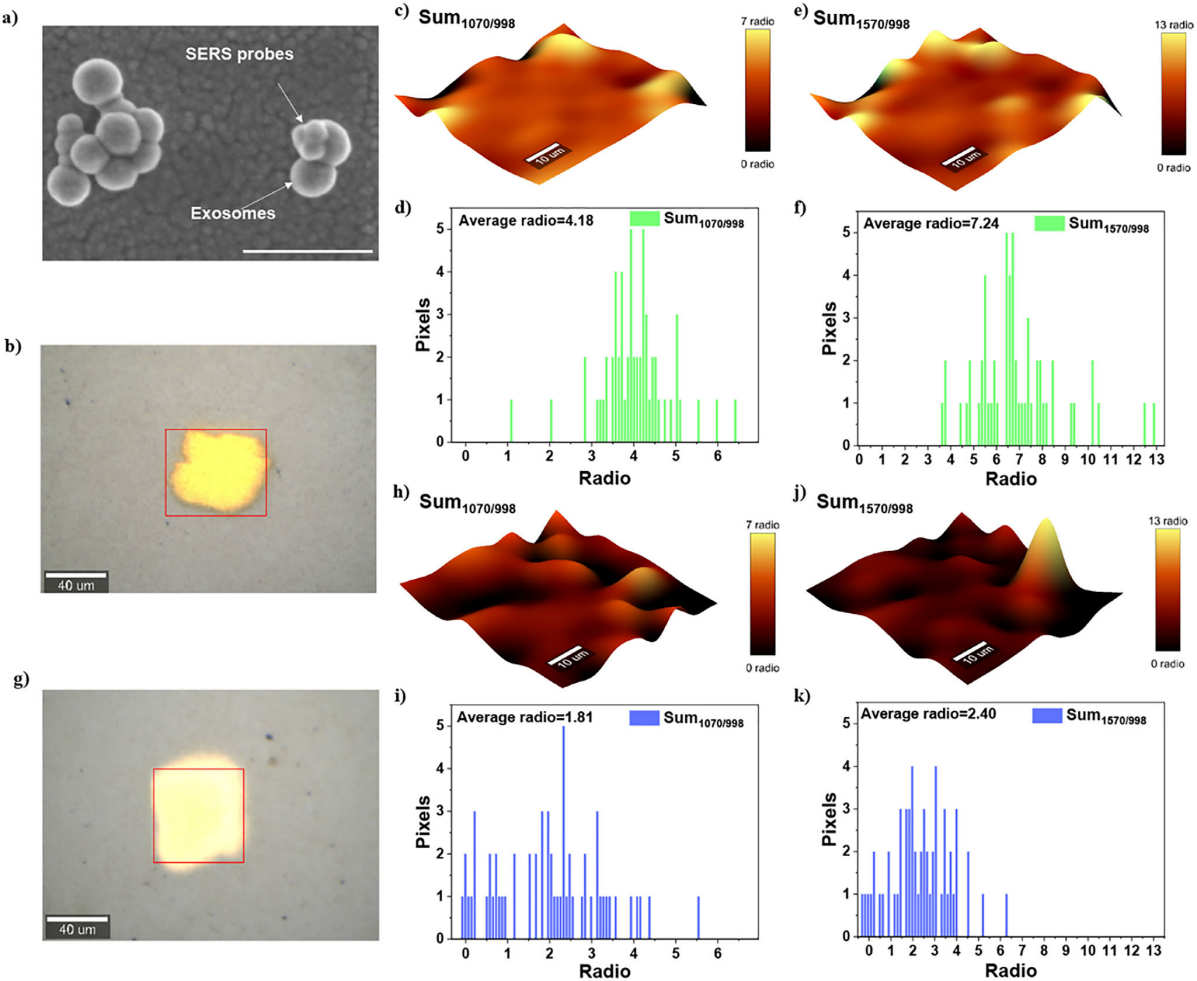
(a) SEM image of exosomes captured in serum using the SERS nanosensors targeting exosomal SA. Scale bar is 400 nm. (b) Bright‐field image corresponding to the SERS mapping image of H individual. (c) SERS mapping image of the sum ratio of peak areas (Sum) at 1070/998 cm^−1^ for SA in H exosomes, along with (d) its image histogram, and (e) the sum ratio of peak areas at 1570/998 cm^−1^ with (f) its image histogram. (g) Bright‐field image corresponding to the SERS mapping image of OC exosomal SA. The red area indicates the acquisition range. (h) SERS mapping image of the sum ratio of peak areas at 1070/998 cm^−1^ for SA in OC exosomes, along with (i) the image histogram. (j) SERS mapping image of the sum ratio of peak areas at 1570/998 cm^−1^ for SA in OC exosomes, along with (k)the image histogram.

After confirming that the AgNP@MPBA nanosensor can detect SA expression, we sought to integrate it with an exosome capture chip to enable subsequent exosome SA detection via SERS. Before this, we evaluated the potential non‐specific adsorption of exosome capture chips to nanosensors. As shown in Figure S8, we observed very little nonspecific binding, confirming that the system is not disturbed by nonspecific adsorption. Subsequently, the chip with captured exosomes was incubated with AgNP@MPBA, and its targeting ability to exosomal SA was confirmed by SEM (Figure 3a). To ensure the stability of the nanosensor after binding with exosomes due to the formation of the MPBA‐SA interaction, we incubated the chip capturing OC serum‐derived exosomes with AgNP@MPBA for 1 h, followed by spectral acquisition at 1 h intervals. As shown in Figure S9, the I_1070_/I_998 cm‐1_ ratio exhibited no significant variation, demonstrating that the nanosensor maintained excellent stability upon exosome binding. Further evaluation of signal uniformity among multiple gold arrays on a single chip was performed (Figure S10A,B). The RSD value for the primary characteristic peak (I_1070_/I_998 cm‐1_) was 6.85% (Figure S10C), indicating excellent signal uniformity between gold arrays.

Based on the above outstanding performance, systematic SERS mapping imaging was performed to visualize the SA expression differences between healthy individuals (H) and the OC group. The representative SERS mapping images (Figure 3b,c,e) and corresponding quantitative analysis of peak area ratios at 1070/998 cm^−1^ (Figure 3d) and 1570/998 cm^−1^ (Figure 3f) were obtained from serum‐derived exosomes of H individuals. Parallel experiments were performed on OC patient‐derived exosomes (Figure 3g–k). Quantitative analysis demonstrates a statistically significant elevation in the mean peak area ratio at 1070/998 cm^−1^ in the H group (4.18) compared to the OC group (1.81) (Figure 3d,i). Similarly, the mean ratio at 1570/998 cm^−1^ is substantially higher in the H group (7.24) than in the OC group (2.40) (Figure 3f,k). These findings provide compelling evidence that exosomal SA expression is significantly upregulated in OC compared to H.

### 2.4 Machine Learning Algorithm‐Assisted SERS Spectroscopy for OC Diagnosis According to Exosomal SA

**FIGURE 4:**
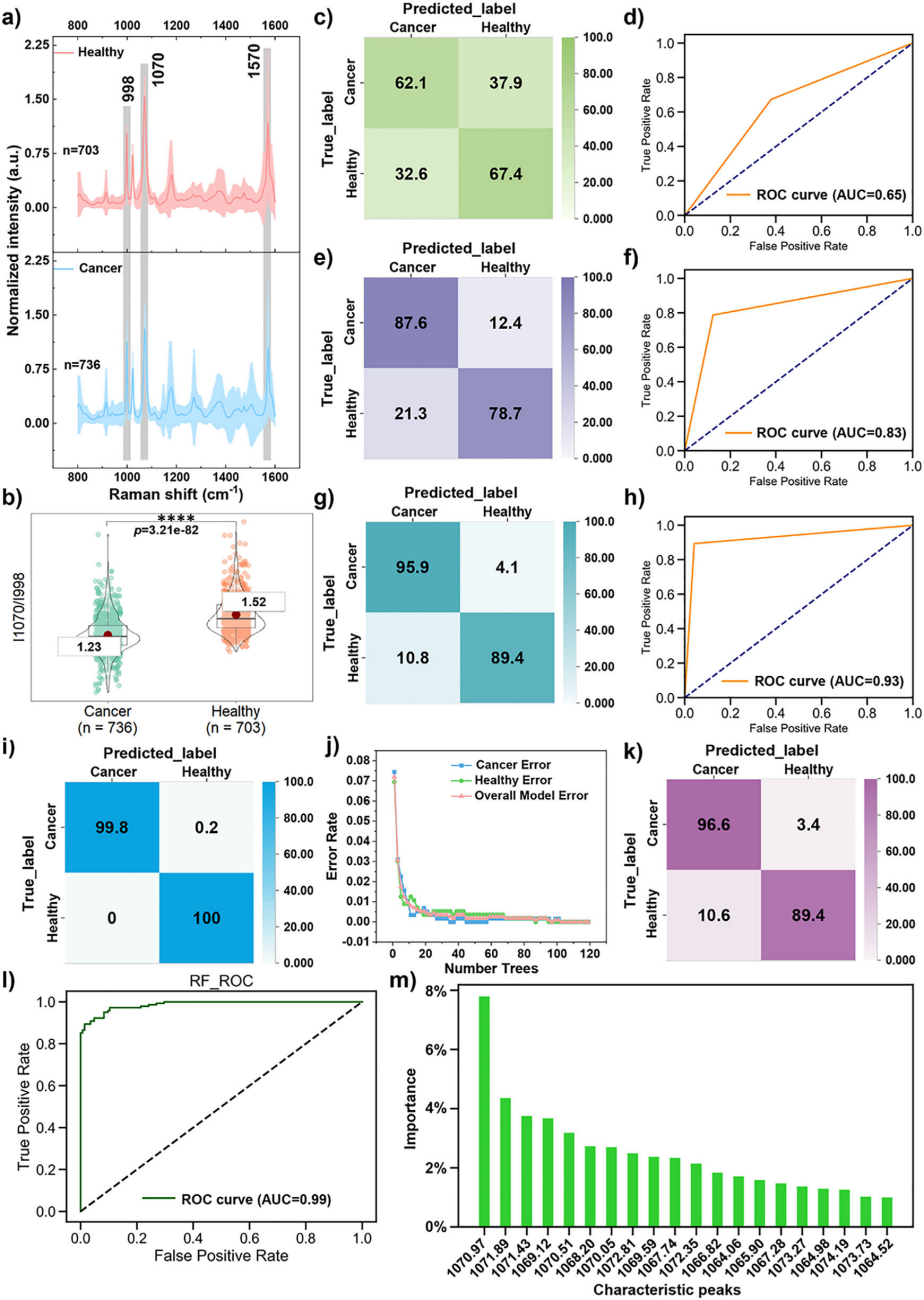
(a) Mean SERS spectra of exosomal SA from OC patients (bottom) and H individuals (top). λex = 632.8 nm, t = 20 s, accumulation = 1 time. The shaded areas represent the standard deviation. (b) Violin scatter plots of I_1070_/I_998 cm‐1_ with *t*‐test results. Each scatter represents one SERS spectrum. ^****^ *p* < 0.0001, ^***^ *p* < 0.001, ^**^ *p* < 0.01, ^*^ *p* < 0.05. (c,d) Test results of the LDA classifier and its ROC curve. (e,f) Test results of the KNN classifier and its ROC curve. (g,h) Test results of the SVM classifier and its ROC curve. (i) The training matrix of the RF classifier. (j) Error rate curve for the RF classifier training set. (k,l) Test results of the RF classifier and its ROC curve. (m) Importance ranking of features in RF classifiers.

Figure 4a shows the mean SERS spectra collected from 20 H individuals and 20 OC patients. Violin plots with *t*‐test analysis revealed significant differences in intensity ratios at I_1070_/I_998 cm‐1_ (Figure 4b) and I_1570_/I_998 cm‐1_ (Figure S11a) between H and OC groups. Notably, the mean intensity ratio of H exceeds that of the OC group, indicating lower average exosomal SA levels in the H group. Figure S11b illustrates the clustering results, which separate H and OC into two distinct groups. Further, heatmaps displaying the mean values for 20 H and 20 OC groups are generated to illustrate individual variation (Figure S11c), revealing noticeable inter‐individual differences. To facilitate the assessment of inter‐group separability, we employed the unsupervised machine learning algorithm *t*‐distributed Stochastic Neighbor Embedding (*t*‐SNE) to visualize high‐dimensional spectra in two dimensions. This visualization reveals clustering patterns and overlapping regions of samples in low‐dimensional space, indicating the limited discriminative capability of *t*‐SNE (Figure S11d).

Therefore, to establish a robust OC diagnostic model, the classifier hyperparameters were optimized via grid search combined with 5‐fold cross‐validation (using validation set accuracy/AUC as the evaluation metric), and a comparative analysis was conducted across multiple machine learning models under the same data splitting strategy. First, the classification performance of the linear discriminant analysis (LDA) algorithm was evaluated. Figure 4c shows that the classification accuracies for OC and H in the test set were 62.1% and 67.4%, respectively. The performance of the test set was analyzed using a ROC curve, yielding an area under the curve (AUC) of 0.65, indicating poor separation capability (Figure 4d). Subsequently, the classification performance of the k‐nearest neighbors (KNN) algorithm was evaluated, revealing classification accuracy rates of 87.6% for OC and 78.7% for H (Figure 4e), with an AUC of 0.83 (Figure 4f), indicating that the performance remained suboptimal. Further evaluation of the support vector machine (SVM) algorithm revealed a classification accuracy of 95.9% for OC and 89.4% for H (Figure 4g). The SVM model achieved an overall recognition accuracy of 92.7% and an area under the curve (AUC) of 0.93 (Figure 4h), indicating exceptional discriminatory performance. Finally, the random forests (RF) algorithm was evaluated. Figure 4i presents the confusion matrix demonstrating training‐set performance, with classification accuracies of 99.8% for OC and 100% for H. Figure 4j further reveals that this classifier exhibits both a low overall error rate and class error rates. Subsequently, the classifier was validated on the test set, demonstrating outstanding performance with a 96.6% recognition rate for ovarian cancer (OC) and 89.4% accuracy for H (Figure 4k). The model achieved an overall classification accuracy of 93% along with an AUC of 0.99 (approaching ideal discriminative performance), confirming the RF model's exceptional classification capability (Figure 4k,l). Notably, the RF model maintains high generalization performance while providing stable, interpretable feature importance and peak contribution rankings. As shown in Figure 4m, the top 20% of peaks by feature importance contribution are all ranked near 1070 cm^−1^, indicating that the B‐OH stretching vibration (1070 cm^−1^) plays a crucial role in OC diagnosis. This aligns with our strategy for OC diagnosis, which evaluates exosomal SA expression using the I_1070_/I_998 cm‐1_ intensity ratio. Based on these results, the RF classifier demonstrates superior classification performance and model interpretability. Consequently, adopting the RF model as a diagnostic tool for OC will enhance clinical understanding and facilitate subsequent target optimization.

### 2.5 Dynamic Monitoring of the Correlation between Exosomal SA and Treatment Response

**FIGURE 5:**
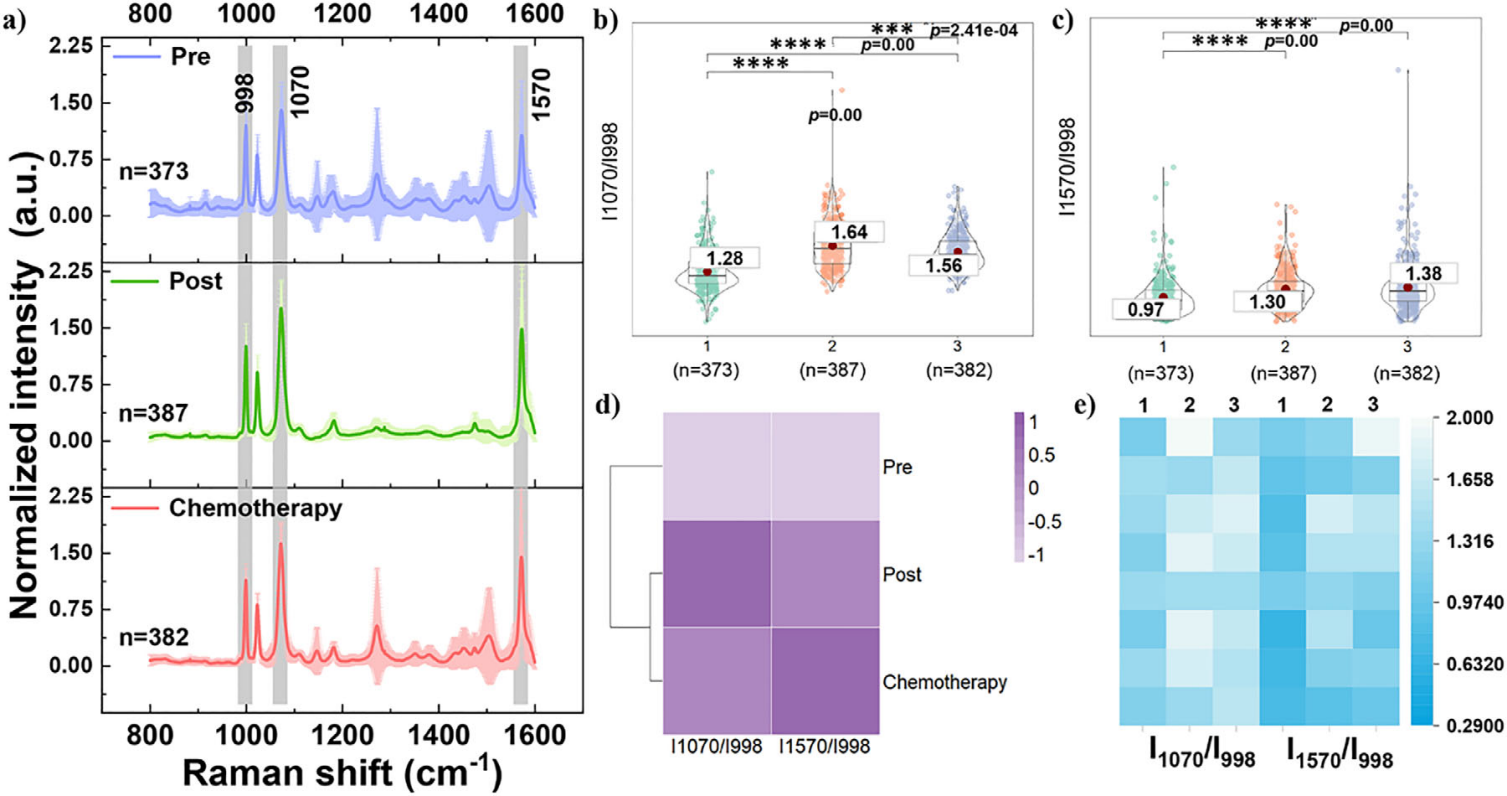
(a) Mean SERS spectra of serum‐derived exosomes from OC patients during preoperative (top), postoperative (middle), and chemotherapy (bottom) periods after 1 h incubation with AgNP@MPBA nanosensors. λex = 632.8 nm, t = 20 s, accumulation = 1 time. The shaded areas represent the standard deviation. (b,c) Violin scatter plot with *t*‐test results at I_1070_/I_998 cm‐1_ and I_1570_/I_998 cm‐1_ during preoperative (1), postoperative (2), and chemotherapy (3) phases of OC, respectively. Each scatter represents one SERS spectrum. (d) Hierarchical clustering heatmap. (e) Heatmaps of mean intensity ratios (I_1070_/I_998 cm‐1_ and I_1570_/I_998 cm‐1_) for eight OC patients, with each strip representing one individual.

The dynamics of SA expression in exosomes from OC patients at different treatment periods were observed, and a comprehensive analysis of data from preoperative, postoperative, and chemotherapy periods was performed. Figure 5a illustrates the mean SERS spectra of SA on exosomes derived from OC patients in these three treatment periods. Assessment of the mean intensity ratios of I_1070_/I_998 cm‐1_ and I_1570_/I_998 cm‐1_ reveals significantly lower values during the preoperative period compared to postoperative and chemotherapy periods (Figure 5b,c), suggesting elevated SA expression on exosomes in the preoperative period. Statistical verification using *t*‐tests confirmed significant differences between preoperative and postoperative periods, as well as between preoperative and chemotherapy periods (Figure 5b,c). Hierarchical cluster analysis (Figure 5d) divides the data into two major clusters, with the preoperative period forming one major category and the postoperative and chemotherapy periods the other. Individual variation analysis reveals significant inter‐individual differences across time points (Figure 5e).

**FIGURE 6:**
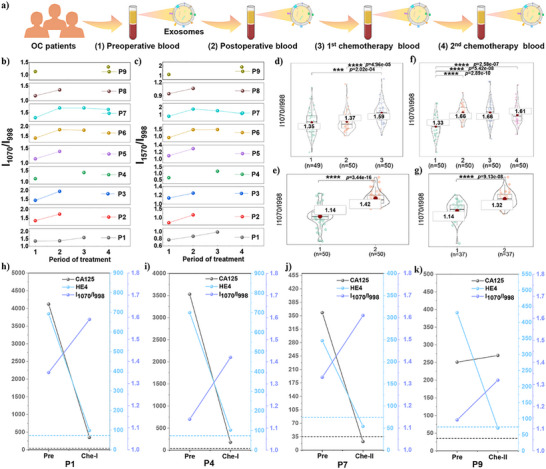
(a) Schematic diagram of the treatment process for OC patients and blood collection time points during preoperative (1), postoperative (2), first chemotherapy (3), and second chemotherapy (4) periods. (b,c) Line graphs showing mean intensity ratios (I_1070_/I_998 cm‐1_ and I_1570_/I_998 cm‐1_) for nine OC patients (P1‐P9) across various treatment periods. (d–g) Violin plots with *t*‐test analyze I_1070_/I_998 cm‐1_, showing the characteristics of P1, P4, P7, and P9 patients during treatment, respectively. (h–k) Line graphs showing CA125 [U/mL] and HE4 [pmol/L] values from clinical tests at different treatment periods for P1, P4, P7, and P9 patients, along with the I_1070_/I_998 cm‐1_ (SA) values. Reference ranges for each clinical biomarker are indicated by colored dashed lines.

The real‐time monitoring of core biomarkers enables the assessment of therapeutic efficacy, which is critical for optimizing personalized treatment strategies and improving clinical outcomes. For this purpose, a longitudinal analysis was first conducted on the SA levels of exosomes in OC patients during preoperative, postoperative, and chemotherapy periods (Figure 6a). As shown in Figure 6b,c, the I_1070_/I_998 cm‐1_ and I_1570_/I_998 cm‐1_ during the postoperative and chemotherapy periods were higher than preoperative levels, indicating that exosome expression levels changed during the treatment period. Since the changes in I_1070_/I_998 cm‐1_ and I_1570_/I_998 cm‐1_ were largely consistent, subsequent analyses were conducted using I_1070_/I_998 cm‐1_ for further analysis. The *t*‐test analysis of the I_1070_/I_998 cm‐1_ across different treatment phases revealed statistically significant differences between preoperative levels and the final monitored treatment phase in P1, P4, P7, and P9 patients (Figure 6d–g). The other five OC patients also showed the same significant difference (Figures S13c, S14c, S16c, S17c, and S19c). Additionally, *t*‐SNE and cluster heatmap analyses of exosome SA levels across different treatment periods for each patient revealed a certain degree of difference (Figures S12–S20). Further analysis is needed to determine whether the differential exosomal SA levels correlate with treatment responses.

Carbohydrate antigen 125 (CA 125) is the most widely used tumor biomarker for OC diagnosis [56, 57]. Additionally, studies have shown that combining CA125 with human epididymis protein 4 (HE4) improves the specificity of OC diagnosis [58, 59]. Due to tissue damage caused by surgery, inflammatory exudation, edema, and fibrosis occur in the surgical area within 1–3 months postoperatively. These benign changes often mimic tumor recurrence on CT imaging, making differentiation challenging. In contrast, fluctuations in tumor markers (CA125 and HE4) in the short postoperative period provide a more direct assessment of residual tumor burden. By approximately 3 months post‐surgery, tissue repair is complete, and inflammatory reactions subside. At this stage, CT scans can clearly identify any residual tumor lesions, preventing misdiagnosis. Therefore, clinical tumor assessment is typically performed after the third chemotherapy cycle, using a combined approach that incorporates CT imaging and tumor marker analysis. As shown in Table S4, these are the tumor assessment results based on the comprehensive evaluation by clinicians using CA 125, HE4, and CT scans following the third cycle of chemotherapy, which stratified patients into complete response (CR) and partial response (PR).

Since patients cannot undergo frequent X‐ray examinations between surgery and the third cycle of chemotherapy, an auxiliary method for assessing residual tumor burden is often required. Numerous studies have demonstrated that elevated or declining CA125 levels correlate with disease progression or regression, indicating its significant clinical value in monitoring treatment response and predicting recurrence in OC patients [60]. Furthermore, dynamic changes in serum CA125 levels during treatment have been linked to therapeutic efficacy, with a rapid decline suggesting a favorable prognosis [61]. For instance, Markman et al. reported that patients with a >50% reduction in serum CA125 after 2 chemotherapy cycles had a more favorable prognosis [62]. Thus, a decline in CA125 levels within two chemotherapy cycles can serve as a predictive marker for treatment response (CR and PR). Based on these findings, we used CA125 as the primary biomarker for predicting treatment efficacy, supplemented by HE4 for dynamic monitoring, thereby improving the accuracy of early response prediction (since decreases in CA125 and HE4 often precede tumor shrinkage observed by imaging). This approach is particularly valuable for patients who cannot undergo frequent radiographic evaluations.

The CA125 and HE4 values measured clinically for each patient during two cycles of chemotherapy are shown in Table S2. Comparing the I_1070_/I_998 cm‐1_ with CA125 and HE4 values, Figure 6h,i shows that during the first chemotherapy period, both P1 and P4 exhibited CA125 decline rates >50% relative to preoperative levels, with HE4 demonstrating a similar trend. However, both remained above normal reference values, suggesting a PR to treatment, consistent with clinical tumor assessment results (Table S4). Concurrently, the exosomal SA expression level, reflected by the I_1070_/I_998 cm‐1_, exhibited a significant reduction with a statistically significant difference between the two treatment periods. Similarly, Figure S17d and Figure 6j demonstrate that in patients P6 and P7, both CA125 and HE4 levels decreased by >50% during chemotherapy compared to preoperative values, falling below the reference range. This indicates a CR, which again aligns with clinical tumor assessment results (Table S4). At this point, the exosomal SA expression levels during the chemotherapy periods for both patients were also significantly lower than pre‐treatment levels, with statistically significant differences. Importantly, the mean exosomal SA level in CR patients was lower than that in PR patients. Figure 6k shows that during the second chemotherapy period, P9 exhibited a 7.5% increase in CA125 levels but an 83.5% decrease in HE4 levels, indicating PR. Clinical evaluation after three chemotherapy cycles confirmed CR (Table S4), validating our prior prediction of PR. At this point, exosomal SA levels were significantly reduced compared to preoperative levels. The integrated results indicate that exosomal SA levels in CR/PR patients exhibit a downward trend compared to preoperative levels. P10 was excluded from the independent analysis because an episode of acute cholecystitis occurred before the initiation of the first chemotherapy cycle. This event had the potential to confound the assessment of treatment efficacy based on monitoring changes in SA over time.

In summary, patients within the second chemotherapy cycle were divided into CR and PR groups based on treatment response predicted by clinical biomarkers (CA125 and HE4). This stratification revealed a potential association between exosomal SA levels and treatment response. Notably, patients exhibiting a significant reduction in exosomal SA levels to low values indicated CR or PR. These findings provide a valuable biomarker‐based framework for guiding personalized treatment strategies.

## 3 Conclusions

This study presents a simple, rapid, and cost‐effective method for on‐site analysis of exosomal SA, facilitating OC diagnosis and therapeutic monitoring. The approach integrates a CD63 aptamer‐functionalized gold array chip for the specific isolation of exosomes, the AgNP@MPBA nanosensor for specific SA detection, and machine learning‐assisted SERS analysis. Compared to traditional ultracentrifugation methods for purifying exosomes, the CD63 aptamer‐modified gold chip enables rapid and specific isolation of exosomes from microliter samples for in situ detection. This approach significantly reduces sample consumption and simplifies cumbersome purification procedures. The observed peak changes of MPBA during assay detection are attributed to dehydration‐condensation interactions between MPBA and SA, resulting in the formation of a six‐membered ring structure. Density functional theory calculations validated this mechanism and exhibited significant correlation with exosomal SA levels. This study evaluated the performance of multiple machine learning algorithms (LDA, KNN, SVM, and RF) for OC diagnosis. Notably, the RF model demonstrated outstanding classification accuracy while offering stable and clinically interpretable feature importance metrics, thereby providing valuable guidance for clinical decision‐making. Furthermore, we longitudinally monitored exosomal SA levels across different treatment phases (preoperative, postoperative, and chemotherapy) and evaluated therapeutic response (CR, PR) using established clinical biomarkers (CA125 and HE4). These analyses reveal a potential correlation between exosomal SA expression and treatment efficacy. In summary, this exosomal SA assay demonstrates robust clinical applicability, enhancing accuracy and reliability through the integration of machine learning for data processing. With further optimization and clinical validation, this technology holds great promise for advancing precision medicine in OC management.

## 4 Experimental Section

### 4.1 Exosome Capture Chip and Evaluation of Its Performance

The preparation of the exosome capture chips involved the thermal evaporation of a gold array film onto silicon using a patterned metal mask, followed by sequential surface functionalization with cysteamine, bis(sulfosuccinimidyl) 3,3'‐dithiobis(propionate) (DTSSP), NH_2_‐CD63 aptamers, and BSA solution. Cysteine amine is grafted onto the gold array film surface via an Au‐S bond. Furthermore, the bis‐sulfo‐NHS esters of DTSSP mediated binding to cysteamine and NH_2_‐CD63 aptamers via amide bond formation at pH 7.0. This enables the modification of CD63 aptamers on the surface of the gold array film. The modified surface was then blocked with BSA solution to prevent non‐specific adsorption.

The exosome capture capability of the chip was systematically validated through scanning electron microscopy (SEM) and fluorescence confocal microscopy. Serum samples (50 µL) from OC patients were diluted 0.5‐fold in PBS buffer (pH 7.4) and incubated with the capture chip in a 2 mL centrifuge tube under co‐incubation for 3 h. After incubation, the chip underwent three PBS rinses to eliminate nonspecific impurities and was nitrogen‐dried for subsequent analysis. SEM imaging compared the chip's surface morphology before and after exosome enrichment. To confirm that the captured particles were indeed exosomes, we detected the transmembrane proteins CD9 and CD63, which are uniquely expressed on exosome membranes to distinguish them from other lipoprotein particles. The chips incubated with or without serum were stained with FITC‐CD9 monoclonal antibody and eFluor 660‐CD63‐mAb monoclonal antibody for 1 h, respectively. After thorough washing with PBS to remove unbound antibodies, the chips were imaged using fluorescence confocal microscopy. λex_CD9_ = 488 nm and λex_CD63_ = 561 nm, λem_CD9_ = 517 nm and λem_CD63_ = 668 nm.

### 4.2 DFT Calculation

Density functional theory (DFT) calculations were performed on the Raman spectra of MPBA, Neu5Ac (the primary constituent of SA), and the MPBA‐Neu5Ac complex. Using Gaussian 16 software at the DFT (B3LYP) level, combined with the B3LYP functional, the 6–31++G(d,p) basis set, and the DFT‐D3 correction term, the ground‐state molecular structures were optimized, and the corresponding vibrational frequencies were calculated. GaussView 6.0 software was used to perform the detailed Raman band assignments.

### 4.3 Human Blood Samples Collection

This study adhered to the guidelines of the Declaration of Helsinki and the Ethics Review Committee of the First Hospital of Jilin University. Human serum samples were obtained from the Department of Biobank, Division of Clinical Research, The First Hospital of Jilin University, and clinical diagnosis and treatment (Ethical Approval No. 25K102‐001). Since the medical records and biological specimens were obtained from previous clinical diagnoses and treatments as well as biobanks, and the patients had already signed a general informed consent form, an application for exemption from informed consent was granted. Exclusion criteria for blood samples, selection of collection time points, and standardized procedures for sample processing are detailed in the Supporting Information. The basic information of OC patients is shown in Table S3.

### 4.4 Statistics

The RStudio software (R4.2.3 environment) was used to perform a *t*‐test analysis of the SERS data. The ‘files2SpectraObject’ function in the ‘ChemoSpec’ package was used to read the data, and the ‘baseline’ package was used for baseline correction. The baseline‐corrected spectra were normalized according to the peak intensity of AgNP@MPBA at 998 cm^−1^. Calculate the values of $I_{1070}/I_{998\;cm^{-1}}\;\mathrm{or}\;I_{1570}/I_{998\;cm^{-1}}$, and use the ‘ggstatsplot’ package to draw violin plots with *t*‐test analysis (two‐sided testing). Finally, obtain the mean values and *P* values. Significance labels were assigned based on *p*‐values: ^****^ represents *p* < 0.0001, ^***^ represents *p* < 0.001, ^**^ represents *p* < 0.01, and ^*^ represents *p* < 0.05.

### 4.5 Machine Learning Data Processing

The Python 3.6 software was used to analyze all SERS data. SERS spectrum preprocessing procedures include spike removal, smoothing and noise reduction, baseline correction, and normalization. Specifically, spike suppression was achieved using a dynamic thresholding approach based on local window median/minima comparison. Subsequently, the spectra underwent Savitzky–Golay smoothing with a third‐degree polynomial and a 7‐point moving window to preserve peak integrity while effectively suppressing high noise. Baseline correction was performed using an adaptive iteratively reweighted penalized least squares (airPLS) algorithm, with the smoothing parameter λ set to 100 and a maximum iteration limit of 15, prioritizing fitting to the lower envelope to remove fluorescence and slow drift effects. Finally, for normalization, the spectra were processed within a narrow window of 995–1005 cm^−1^, using the peak at 998 cm^−1^ as the reference, applying maximum value normalization to set this peak to 1, ensuring consistent scaling across samples.

To ensure the classification is universally applicable across different populations and disease types, SERS spectra were randomly divided into training and testing sets at an 80:20 ratio without stratification by age or pathological subtype. Specifically, 591 spectra from OC patients were used for the training set, and 145 spectra for the test set. For healthy individuals, 562 spectra were used as the training set and 141 spectra as the test set. To avoid information loss due to prior feature reduction, the full spectrum was used as input without preliminary feature selection. For visualizing class separability (not for training purposes), *t*‐SNE was employed to project high‐dimensional spectral data into 2D space via nonlinear manifold embedding, revealing cluster structures and overlapping regions in the low‐dimensional representation.

The hyperparameters for all classifiers were optimized using grid search combined with 5‐fold cross‐validation (using validation set accuracy/AUC as the criterion). All classifiers reported accuracy, confusion matrices, and ROC curves/AUC values. A comparative analysis was conducted across multiple models (SVM, LDA, KNN, RF) under identical data splits and evaluation metrics. SVM: Kernel function (linear, poly, rbf, sigmoid) selected via grid search with 5‐fold cross‐validation; one‐vs.‐rest decision function. LDA: Also evaluated using cross‐validation. KNN: Number of neighbors, weight function, leaf size optimized via grid search. RF: The key hyperparameters were selected by grid search combined with 5‐fold cross‐validation, including n_estimators (100/200/300), max_depth (5/10/15), min_samples_split (2/4/6), and random_state was fixed to ensure reproducibility. The optimal combination and its validation set performance are reported. The key contributing wavenumbers were subsequently identified through RF model‐based feature importance analysis (using gain/impurity metrics) to ensure interpretability and facilitate potential transfer learning. The code link is https://github.com/lixinli2017/machine‐learning‐models.git.

## Conflicts of Interest

The authors declare no conflicts of interest.

## Supporting information




**Supporting File**: advs73964‐sup‐0001‐SuppMat.docx.

## Data Availability

The data that support the findings of this study are available from the corresponding author upon reasonable request.
