# Supplementary material for: Machine Learning‐Enhanced Analysis of Exosomal Surface Sialic Acid Using Surface‐Enhanced Raman Spectroscopy for Ovarian Cancer Diagnosis and Therapeutic Monitoring
Source: Adv Sci (Weinh). 2026 Jan 21;13(18):e18190. doi: 10.1002/advs.202518190 (PMC13042971; doi:10.1002/advs.202518190)
Supplement: Supplementary file 1 — Supporting File: advs73964‐sup‐0001‐SuppMat.docx. [file ADVS-13-e18190-s001.docx]

**Supporting Information for**

**Machine Learning-Enhanced Analysis of Exosomal Surface Sialic Acid Using Surface-Enhanced Raman Spectroscopy for Ovarian Cancer Diagnosis and Therapeutic Monitoring**

*Lili Cong^1,2,3^, Jiaqi Wang^2^,* *Sijun Huang^4^, Xiaxia Man^1^, Yi Guo^4^, Shuping Xu***^2^, and Songling Zhang***^1,3^*

*^1^* Department of Gynecological Oncology, Gynecology and Obstetrics Center, The First Hospital of Jilin University, Changchun 130021, P. R. China

*^2^* State Key Laboratory of Supramolecular Structure and Materials, College of Chemistry, Jilin University, Changchun 130012, P. R. China

*^3^* Jilin Provincial Key Laboratory of Women’s Reproductive Health, Changchun 130021, P. R. China

*^4^* Key Laboratory for Molecular Enzymology and Engineering, Ministry of Education, School of Life Sciences, Jilin University, Changchun 130012, P. R. China

**AUTHOR INFORMATION**

**Corresponding Authors**

* Department of Gynecological Oncology, Gynecology and Obstetrics Center, The First Hospital of Jilin University, Changchun 130021, P. R. China
E-mail: [slzhang@jlu.edu.cn](mailto:slzhang@jlu.edu.cn) (S. Z.)

* State Key Laboratory of Supramolecular Structure and Materials, College of Chemistry, Jilin University, Changchun 130012, P. R. China

E-mail: xusp@jlu.edu.cn (S. X.)

**Contents**

[Contents 2](#_Toc217376821)

[1. Materials 3](#_Toc217376822)

[2. Instruments 3](#_Toc217376823)

[3. Blood Sample Exclusion Criteria and Selection of Collection Timing 4](#_Toc217376824)

[4. Blood Sample Procedure 4](#_Toc217376825)

[5. Ultracentrifugation Procedure 5](#_Toc217376826)

[6. Preparation of Exosome Capture Chips 5](#_Toc217376827)

[7. Preparation of SERS Nanosensors 6](#_Toc217376828)

[8. Characterization of SERS Nanosensors 6](#_Toc217376829)

[9. Evaluation of SERS Nanosensor Specificity for SA by SEM Imaging and SERS Detection 7](#_Toc217376830)

[10. SERS Mapping Imaging 7](#_Toc217376831)

[11. SERS Data Collection 7](#_Toc217376832)

[12. Figures 9](#_Toc217376833)

[13. Tables 21](#_Toc217376834)

[14. References 25](#_Toc217376835)

1. **Materials**

N-Acetylneuraminic acid, 4-Mercaptophenylboronic Acid (≥90%, contains varying amounts of Anhydride), cysteamine, glucose, galactose, and mannose were bought from Sigma-Aldrich (USA). FITC-CD9 Monoclonal Antibody (eBioSN4 (SN4 C3-3A2)) and eFluor^TM^ 660-CD63 Monoclonal Antibody (H5C6) were purchased from Thermo Fisher Scientific (USA). Silver nitrate (AgNO_3_, >99.8%) was obtained from Beijing Chemical Factory (China). Trisodium citrate dehydrate (99%) was purchased from Shanghai Chemical Reagent Co., Ltd (China). Gold particles (5N, 99.999%) were purchased from Hangzhou Hangdan Optoelectronic Technology Co., LTD (China). Stainless steel metal mask plate processed by the precision mask plate processing center (China). The sequence of the CD63-NH_2_ aptamer was CAC CCC ACC TCG CTC CCG TGA CAC TAA TGC TAT TTT TTT TTT-(CH2)7-NH_2_ 3′. The aptamer was purchased from IBSBIO (China).

1. **Instruments**

An ultra-high-resolution field-emission scanning electron microscope (SEM, Regulus 8100, Hitachi, Japan) was used to capture the morphologies. A preparative ultracentrifuge (Optima L100XP, USA) was used to obtain exosomes *via* the ultracentrifugation method. A ZHD-300M2 high-vacuum resistance evaporation coating system (Beijing Technol Science Co., Ltd., China) was used for depositing gold array films on silicon wafers. SERS spectra were collected using a confocal Raman system (LabRAM Aramis, Horiba JobinYvon, USA). SERS mapping imaging was collected using a confocal Raman imaging system (alpha300 R, Witec, Germany). UV-visible absorption spectra were determined by a UV-visible spectrometer (UV-vis, Ocean Optics USB4000). Confocal fluorescence microscopy (Nikon AX, Japan) was used to image CD9 and CD63 proteins on the surface of exosomes. Dynamic light scattering (Zetasizer Pro, Malvern Panaco, UK) for zeta potential determination. White light interferometric 3D topography measurement system (AM-7000, Atometrics, China) for determining the height and roughness of gold array films vaporized on silicon wafer surfaces.

1. **Blood Sample Exclusion Criteria and Selection of Collection Timing**

Blood samples were collected from ovarian cancer patients (aged 18-75 years) undergoing surgery at the Department of Gynecologic Oncology, First Hospital of Jilin University. Among them, Patients receiving neoadjuvant chemotherapy, splenectomy, contraindication to radiotherapy and chemotherapy, vaccination within 2 months, history of blood transfusion within 2 weeks, immunosuppressive therapy within 2 weeks, long-term use of recombinant human erythropoietin, recombinant human interleukin and other drugs affecting blood cell components, severe organ dysfunction, and premalignant lesions of the blood system were excluded from the sample.

Since OC patients are discharged the day after completing chemotherapy. Therefore, for the monitoring phase of the study, blood samples were obtained 3 to 5 days after surgery (tumor cytoreductive surgery ) and the day after the end of each dose of chemotherapy (paclitaxel and carboplatin). The time range of sample collection, including the interval between surgery and the first chemotherapy (19-38 days) and the interval between two chemotherapies (21-29 days), needs to be dynamically collected according to the actual situation of patients. Since each patient was monitored independently, it was only necessary to ensure that blood samples were taken at each treatment node.

1. **Blood Sample Procedure**

To ensure the consistency of the test, the blood samples were collected into the coagulant tubes for standardized processing. First, the blood samples were centrifuged at 4 °C (1570 rpm, 10 min), and the upper layer of serum was sucked into a clean 2mL centrifuge tube. After further centrifugation (13200 rpm, 10 min), the supernatant was carefully aspirated into a new centrifuge tube for fractionation and stored in a refrigerator at -80 °C. This process can ensure the uniformity of the sample preparation stage to maintain the consistency of the subsequent surface-enhanced Raman spectroscopy detection conditions.

1. **Ultracentrifugation Procedure**

First, dilute 50 μL of serum with PBS to a specific volume. Next, centrifuge at 2000 g for 15 min at 4 °C, remove the supernatant, and resuspend in PBS solution. Under the same conditions, centrifuge at 10000 g for 30 min, remove the supernatant, and resuspend in PBS solution again. Finally, centrifuge at 110,000 g for 70 min. Remove the supernatant and retain the pellet to obtain the exosomes.

1. **Preparation of Exosome Capture Chips**

Silicon sections (2.0 mm × 3.0 mm) were placed in deionized water, ethanol, acetone, ethanol, and deionized water for ultrasonic cleaning for 5 min each. The cleaned silicon slices were blown dry with pure nitrogen. Preparation of gold array patterns on silicon substrates was achieved using the ZHD-300M2 high-vacuum resistance evaporation coating system (Beijing Technol Science Co., Ltd., China) through a precisely controlled thermal evaporation process. Specifically, the silicon wafers are firmly fixed on the surface of a metal mask plate with a periodic pattern (Figure S1a-b). Herein, the width and height of each gold array point are both 60 µm, and the center-to-center distance between adjacent gold array points is 500 µm (Figure S1c). The sample was then transferred into a vacuum deposition chamber. The vacuuming procedure was performed by sequentially starting the mechanical pump and the front-stage valve until the pressure reached 5.00 Pa. Subsequently, the molecular pump was engaged at full rotation speed (704 rpm) until the system achieved the required deposition pressure. Following a 2 h vacuum stabilization period, the evaporation power supply was activated. The deposition of a uniform gold film array onto the silicon substrate was completed via vacuum thermal evaporation at a deposition rate of 0.05 nm/s.

Subsequently, the gold array film chips were immersed in a 1.5 mL centrifuge tube containing 100 µL of cysteamine solution (0.5 mM in ethanol) for 24 h at room temperature, followed by rinsing with PBS solution (pH 7.4) to remove unbound molecules. Then, the silicon slice was incubated in 50 µL of an aqueous bis(sulfosuccinimidyl) 3,3'-dithiobis(propionate) (DTSSP) solution (0.4 mg/mL) with shaking at room temperature for 30 min, followed by three washes with PBS to remove excess DTSSP solution. Next, the NH_2_-CD63 aptamer was incubated with the silicon composite substrate for 30 min at room temperature with shaking, followed by further incubation at 4 °C overnight to modify the CD63 aptamer on the chip surface. Finally, the chip was immersed in a BSA solution (0.05 mg/mL) to seal unmodified sites and prevent nonspecific adsorption, thereby obtaining an exosome capture chip.

1. **Preparation of SERS Nanosensors**

AgNPs were first prepared by the Lee synthesis method.^1^ Specifically, 0.018 g of silver nitrate was taken, then 100 mL of deionized water was added, 2 mL of dissolved sodium citrate (1 wt%) was added immediately after heating to microboiling, and kept at 90 °C for 40 min until the color changed to grayish-green. Finally, the prepared silver nanoparticles were cooled to room temperature and stored in a refrigerator at 4 °C for later use. SEM characterized the morphology. Subsequently, the Ag@MPBA sensing nanoprobes were obtained by surface-modifying the AgNPs with MPBA. Briefly, 3 μL of MPBA (1.0 mM in ethanol) was added to 6 mL of AgNPs (1.89×10^-11^ mol/L) and stirred for 6 h at room temperature. Free MPBA was removed by centrifugation (5600 rpm, 7 min). Finally, the mixture was redispersed in deionized water to prepare Ag@MPBA at a concentration of 1.0×10^-10^ mol/L for subsequent experiments. Uv-vis spectroscopy, dynamic light scattering and Raman spectroscopy were further used to characterize the obtained SERS sensing nanoprobes.

1. **Characterization of SERS Nanosensors**

Firstly, AgNPs were prepared, and their morphology and dimensions are shown in Figure S6a. The particle size distribution was 56 ± 5 nm, with an RSD of 8.1%, indicating that the synthesized AgNPs had relatively homogeneous properties (Figure S6b). Secondly, MPBA molecules were modified on the surface of AgNPs to prepare SERS sensing probes specifically responsive to exosomal sialic acid. The UV-visible absorption spectrum shows that the maximum absorption wavelength of AgNPs increased by 4 nm after modifying the MPBA molecules (Figure S6c). Meanwhile, the zeta potential of the prepared nanoprobes increased by 6.51 mV (Figure S6d). In conclusion, it is proven that the SERS sensing probes were successfully prepared. The stability of the prepared SERS sensing probes was then evaluated, and the RSD values of the statistical SERS sensing probes at I_1070_/I_998 cm-1_ and I_1570_/I_998 cm-1_ were 6.7% and 10.4%, respectively (Figure S6e-f). This indicates that the probes are stable, with the I_1070_/I_998_ _cm-1_ exhibiting better stability.

1. **Evaluation of SERS Nanosensor Specificity for SA by SEM Imaging and SERS Detection**

As mentioned above, after the capture exosome chip was separated and enriched with serum exosomes, it was washed three times with Tris-HCl buffer (pH 7.4). Then, 50 µL AgNP@MPBA was added and co-incubated with the capture chip for 1 h. The chip was then washed three times with Tris-HCl buffer (pH 7.4) to remove any unbound nanoprobes, followed by SEM imaging characterization. Meanwhile, SERS detection was carried out after incubating glucose (1 mM), galactose (1 mM), mannose (1 mM), and SA (1 mM) with AgNP@MPBA for 1 h to further evaluate the specificity of SERS sensors. λex=632.8 nm, t=2 s and accumulation=1 time.

1. **SERS Mapping Imaging**

The exosome capture chip was used to capture and enrich exosomes from the serum of healthy individuals and ovarian cancer patients, respectively. After that, the capture chip was rinsed three times with Tris-HCl buffer (pH 7.4) and then incubated with 50 µL of AgNP@MPBA for 1 h. Subsequently, the chip was rinsed three times with Tris-HCl buffer (pH 7.4) to remove the unbound nanoprobes. Finally, the chip was placed in a glass-bottomed culture dish containing PBS solution for SERS mapping imaging. λex=633 nm, t=5 s, accumulation=1 time and laser power =5 mW. SERS mapping imaging parameters were set to acquire 8 points per line and 8 lines per image.

## **SERS Data Collection**

All serum samples were diluted 0.5-fold in PBS buffer (pH 7.4) and incubated with the exosome capture chip in a 2 mL centrifuge tube by gentle shaking for 3 h. The chip was then washed three times with Tris-HCl buffer (pH 7.4, 10 mM) to remove excess unbound serum impurities. Next, the chip was incubated with 50 μL AgNP@MPBA nanoprobes for 1 h, followed by three rinses with Tris-HCl buffer to remove unbound nanoprobes. Finally, the chip was placed in a glass-bottomed culture dish containing PBS buffer for SERS measurement. A confocal Raman system equipped with a 7 mW HeNe laser (LabRAM Aramis, Horiba Jobin-Yvon) was used to collect SERS spectra from serum samples. For SERS spectral acquisition, we collected 736 spectra from patient samples (P1-P20) with the number of spectra per sample set as follows: 49, 50, 45, 50, 49, 49, 50, 50, 37, 38, 39, 45, 42, 23, 20, 20, 20, 20, 20, and 20 spectra, respectively. The healthy control group (H1-H20) yielded a total of 703 spectra, with the number of spectra per sample group as follows: 48, 47, 55,54, 48, 49, 30, 31, 30, 31, 30, 30, 30, 20, 25, 30, 30, 30, 25, and 30 spectra, respectively. All acquired spectra (totaling 1,439) were included in the machine analysis without any exclusion. Additionally, SERS spectra were collected from nine OC patients during preoperative, postoperative, and chemotherapy periods. All SERS spectra were recorded at 632.8 nm with an acquisition time of 20 s per measurement using a 50× objective lens, following calibration with a 520.7 cm^-1^ silicon wafer before measurement.

1. **Figures**


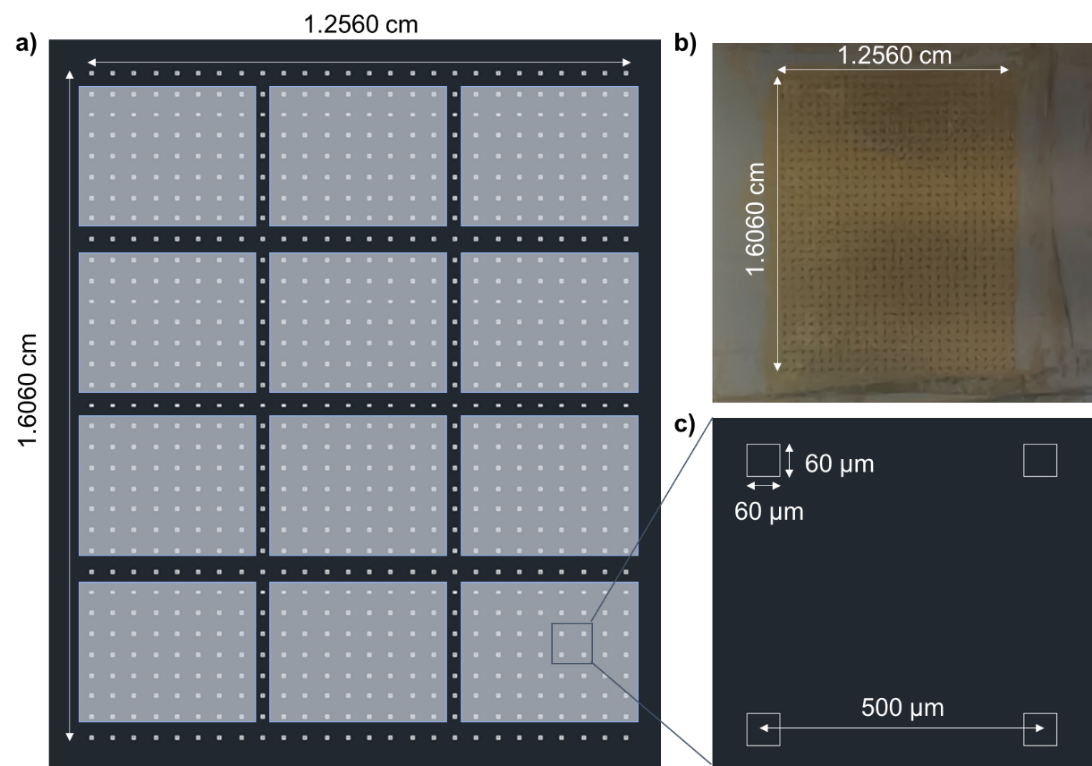


Figure S1. (a) Design drawing of the metal mask. Dark color represents the metal mask, while light gray indicates multiple silicon wafers. (b) The physical drawing of a metal mask. (c) Schematic representation of local amplification of a metal mask.


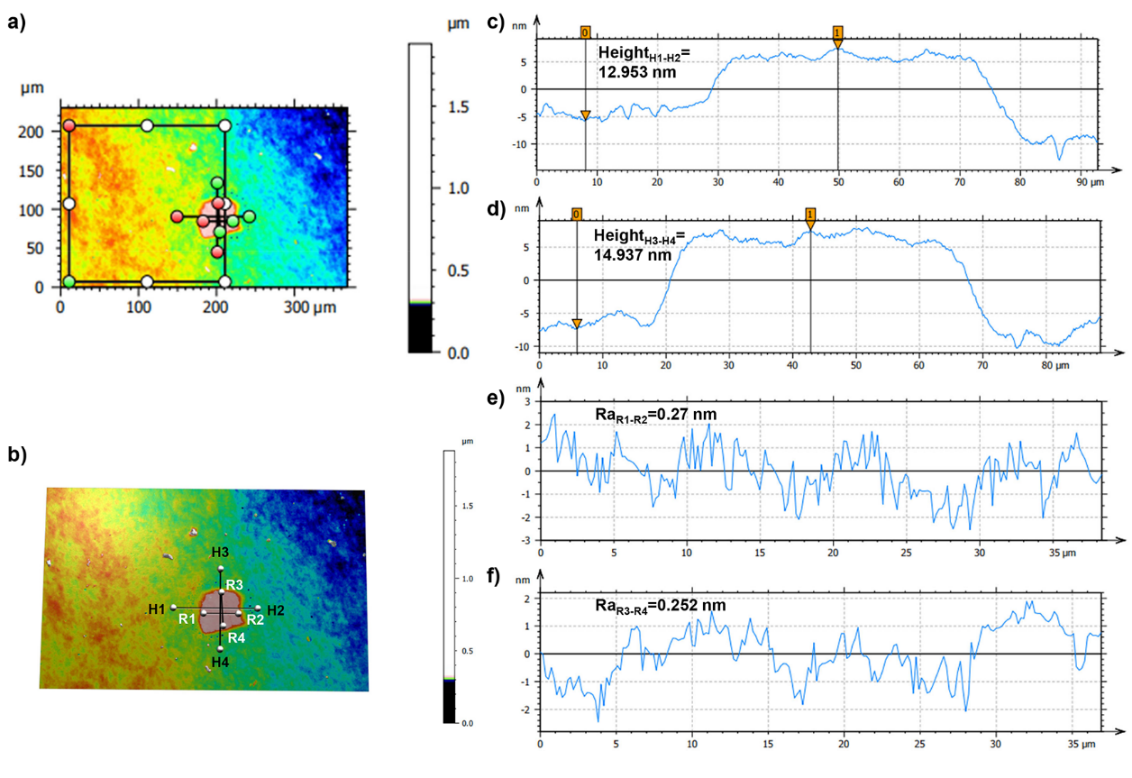


Figure S2. (a) Display of the measurement area of silicon-gold array films. (b) The sampling lengths for height and roughness measurements. H represents the length for height measurement, and R represents the length for roughness measurement. (c)-(d) Measuring the height of the gold film in the H1-H2 and H3-H4 directions, respectively. The measurement result is the marked distance of 0 to 1. (e)-(f) Measuring the roughness of the gold film in the R1-R2 and R3-R4 directions, respectively.


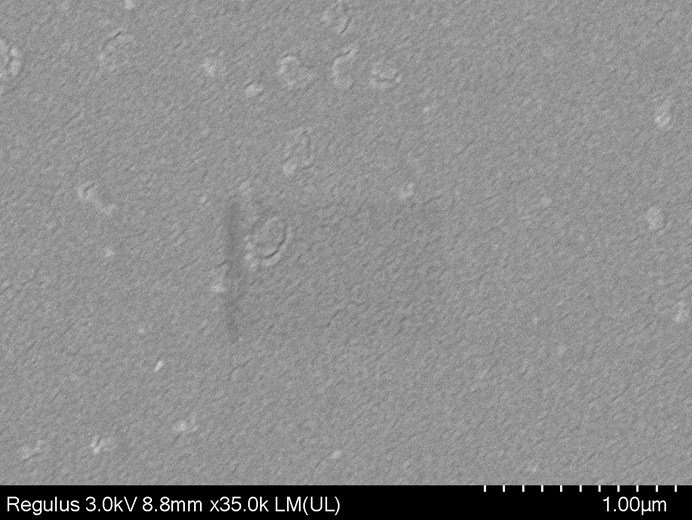


Figure S3. SEM images of exosome capture chips without assembled CD63 aptamer incubated with serum for 3 h. Scale bar is 1 µm.


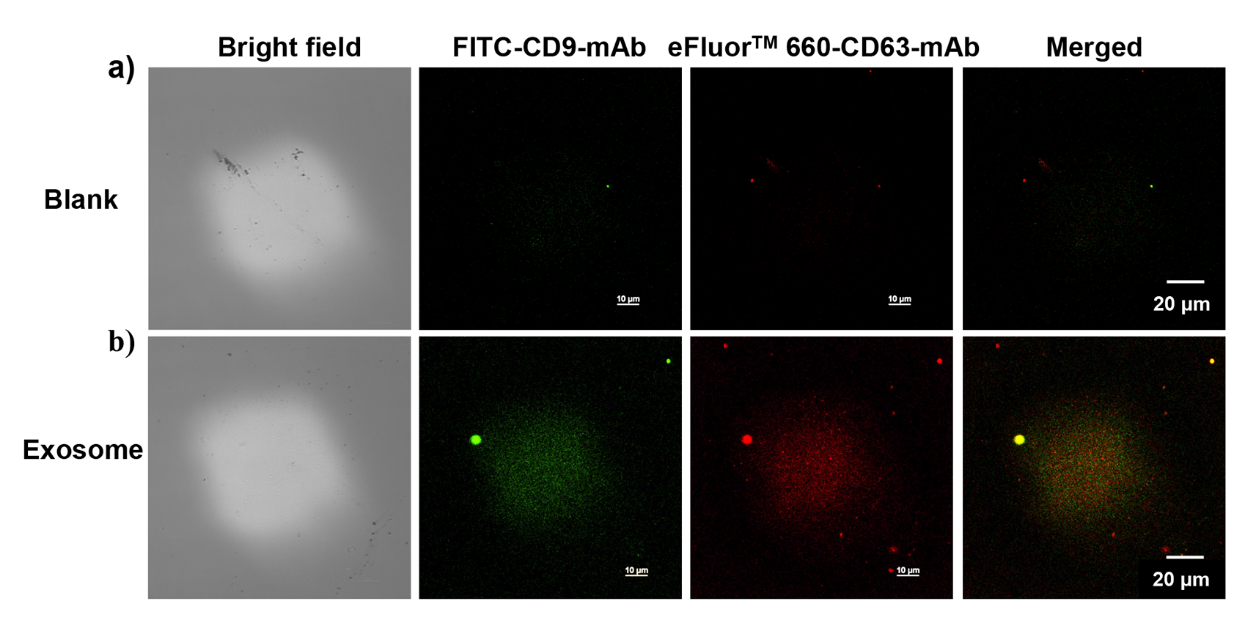


Figure S4. Bright-field, CD9, CD63 and merged images of capture exosome chip without (a) or with (b) exosomes. λex_CD9_=488 nm and λex_CD63_=561 nm, λem_CD9_=517 nm and λem_CD63_=668 nm. Scale bar represents 20 μm.


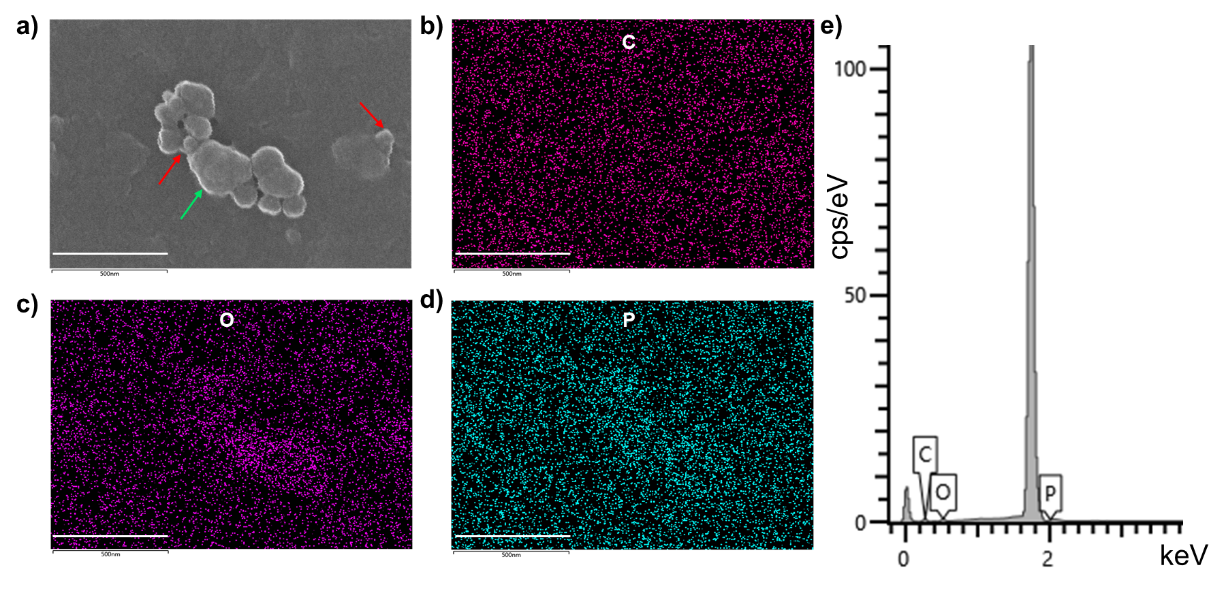


Figure S5. (a) SEM image of serum-derived exosomes purified by ultracentrifugation, along with corresponding elemental mapping images of C (b), O (c), and P (d). Green and red arrows indicate co-precipitated lipoproteins or large vesicles and broken vesicle membranes, respectively. Scale baris 500 nm. (e) Energy spectrum of ultracentrifugation-purified exosomes.


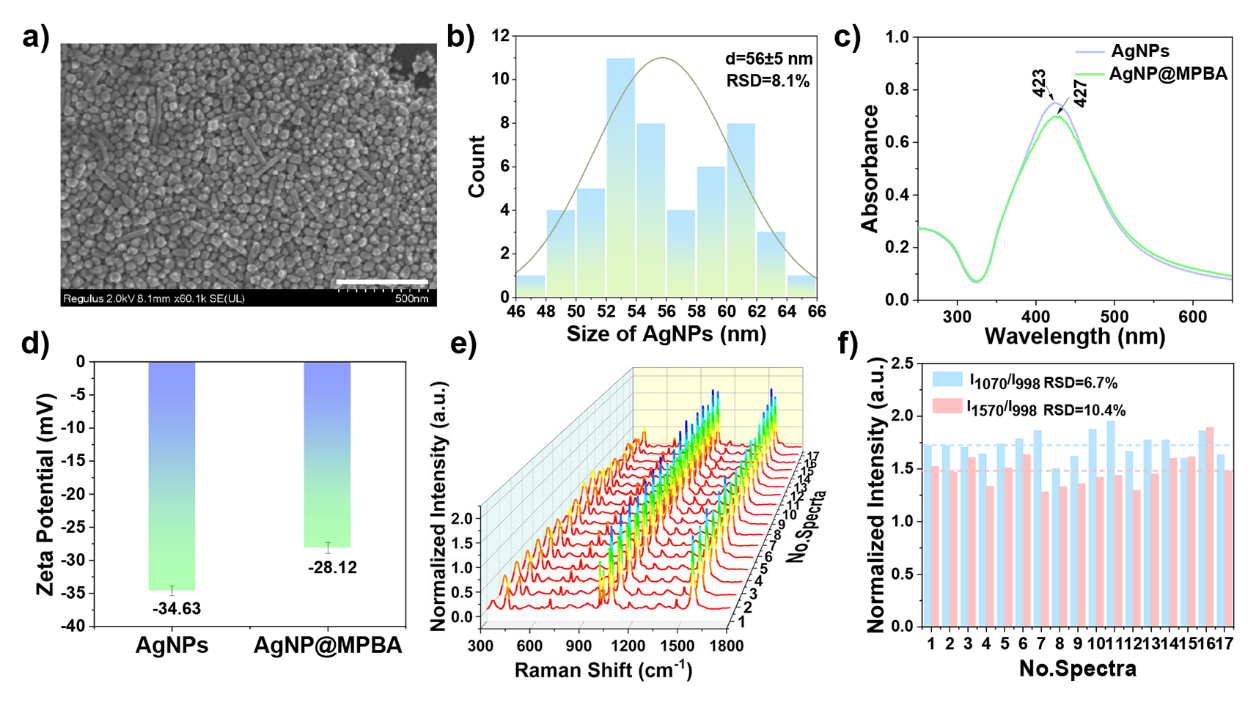
Figure S6. (a) SEM images of AgNPs. Scale bar is 500 nm. (b) Size distribution of AgNPs particles. (c) UV-vis spectra of AgNPs and AgNP@MPBA. (d) The zeta potential of AgNPs and AgNP@MPBA. Error bars represent the standard deviation. (e) SERS stability determination of AgNP@MPBA nanoprobes. (f) Homogeneity results for AgNP@MPBA nanoprobes. The intensity of I_1070_/I_998_ and I_1570_ /I_998_ _cm-1_ was plotted from 17 spectra from (e).


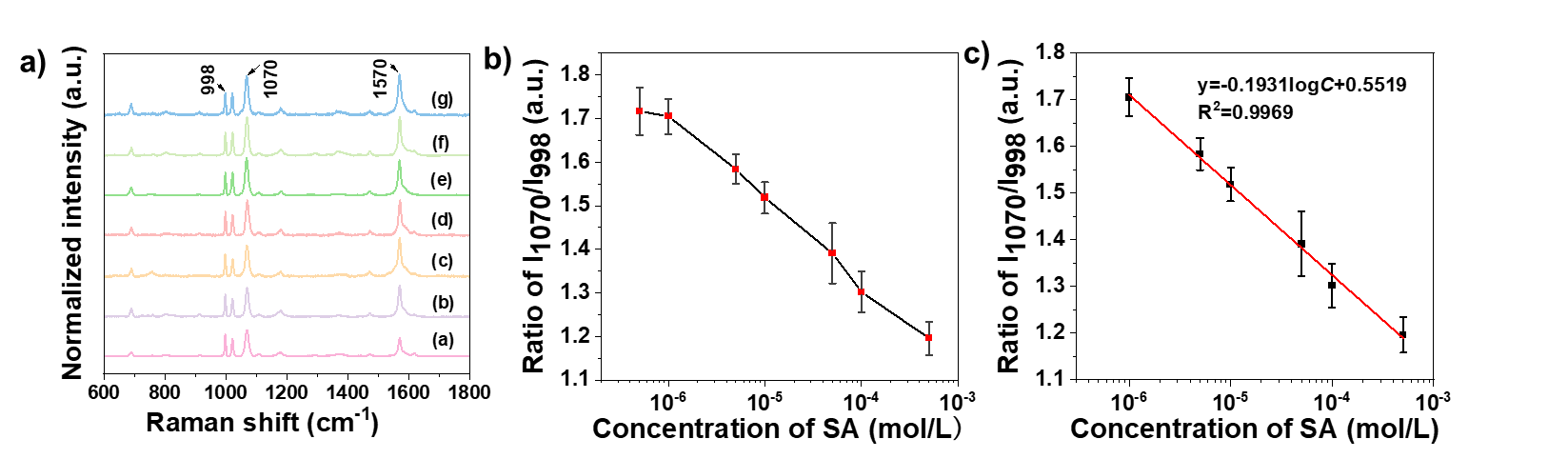


Figure S7. (a) The mean SERS spectra of AgNP@ MPBA incubated with SA solutions of different concentrations (from a to g: 5.0×10^-4^ mol/L, 1.0×10^-4^ mol/L, 5.0×10^-5^ mol/L, 1.0×10^-5^ mol/L, 5.0×10^-6^ mol/L, 1.0×10^-6^ mol/L, 5.0×10^-7^ mol/L) for 1 h in Tris-HCl buffer (pH 7.4), respectively. The mean SERS spectrum is obtained from three SERS spectra. λex=632.8 nm, t=2 s, accumulation=1 time. (b) The SA detection curve in Tris-HCl was obtained by plotting the mean intensity ratio of I_1070_/I_998_ _cm-1_ as a function of the SA concentration after incubation for 1h. Error bars represent the standard deviation. (c) The standard curve for detecting different concentrations of SA. Error bars represent the standard deviation.


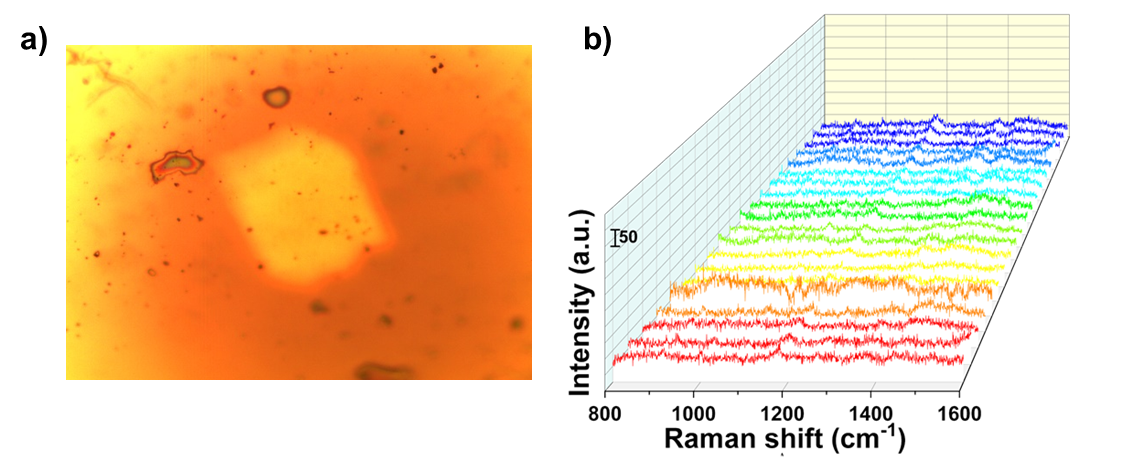


Figure S8. Bright-field imaging (a) and SERS spectra (b) of a silicon-gold array film-CD63 aptamer chip incubated with AgNP@MPBA for 1 h. λex=632.8 nm, t=20 s, accumulation=1 time.

Figure S9. The mean SERS spectrum was collected at 1h intervals after the nanosensor was combined with exosomes. The first collected SERS spectra were used as the control group. The mean SERS spectrum is obtained from three SERS spectra. λex=632.8 nm, t=20 s, accumulation=1 time. The shaded areas represent the standard deviation.


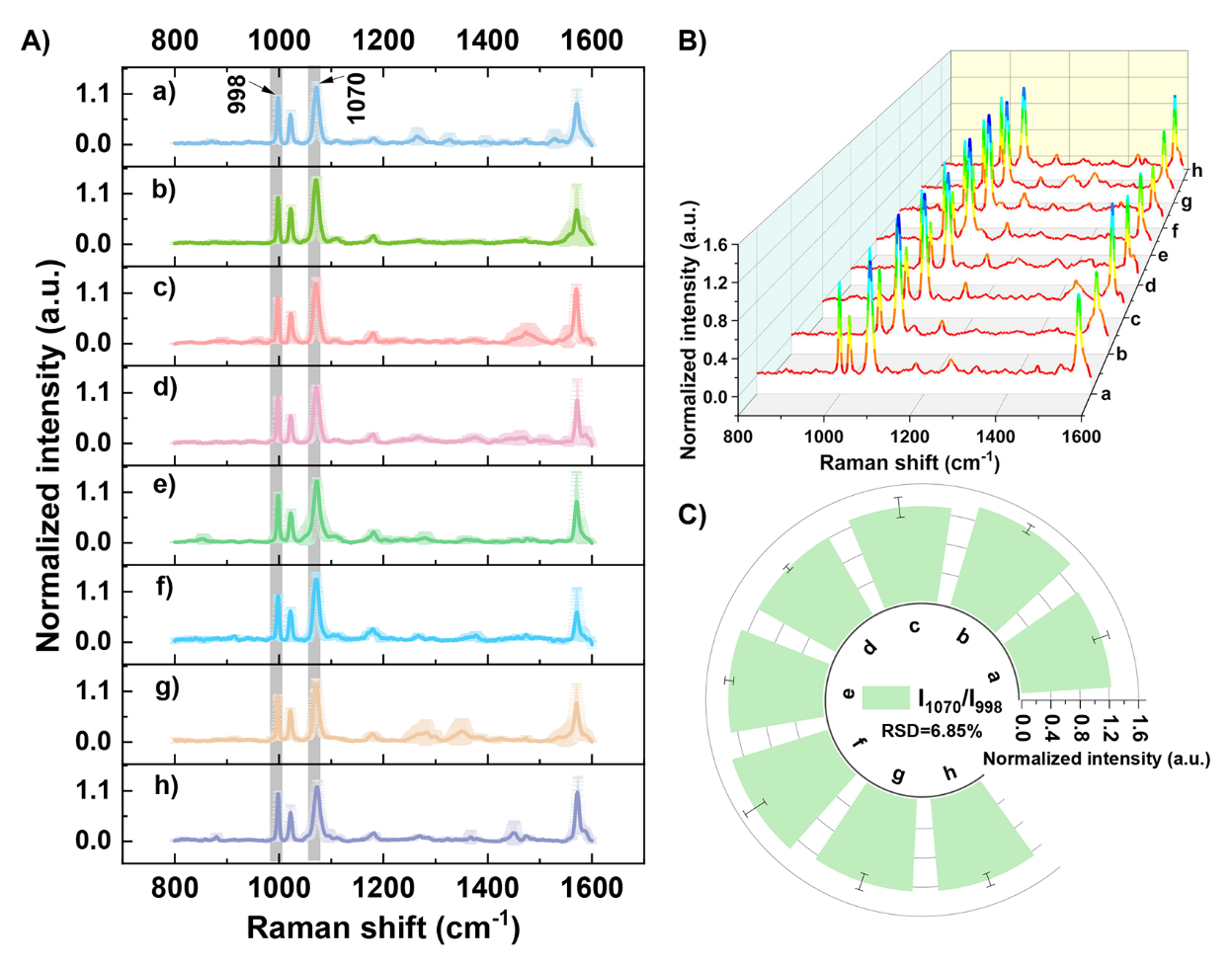


Figure S10. (A) Mean SERS spectra for each gold array (a-h) after co-incubation of the OC-derived exosome capture chip with AgNP@MPBA. Mean spectra derived from three randomly selected SERS spectra per gold array. The shaded regions of the spectrum represent the standard deviation. (B) Color-mapped 3D waterfall plot of the average spectrum of the (a-h) gold array. (C) Evaluation of signal uniformity at the primary characteristic peaks (I_1070_/I_998_ _cm-1_) in the average spectra of multiple (a-h) gold arrays. Error bars represent the standard deviation.


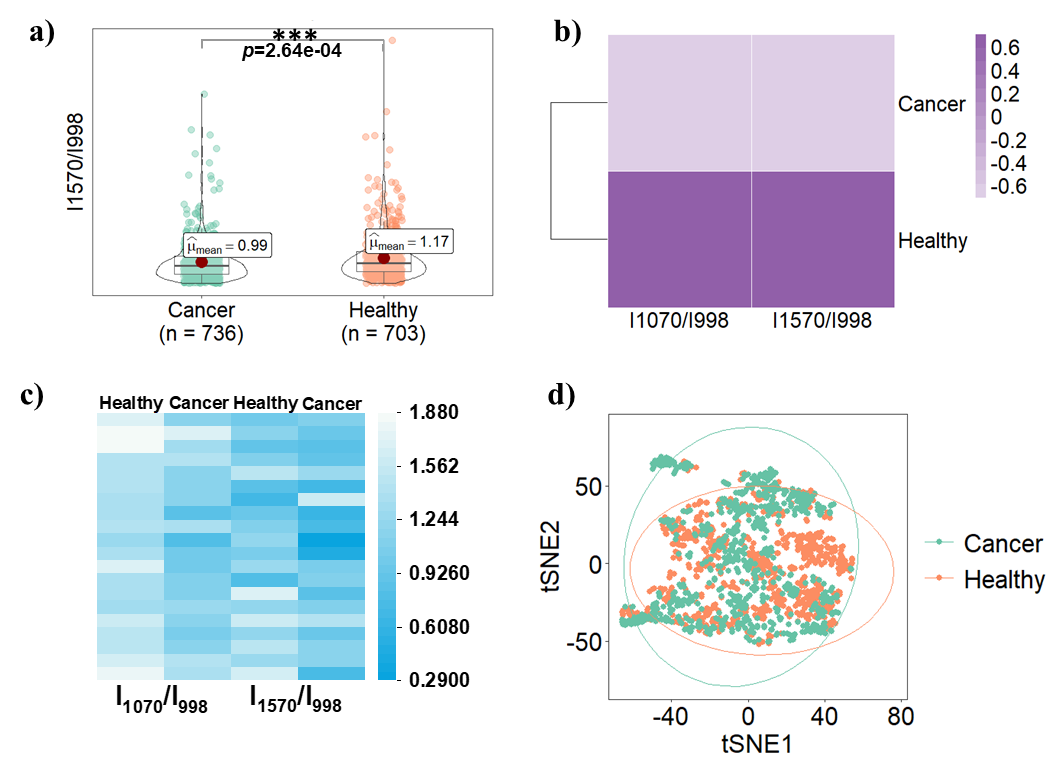


Figure S11. (a) Violin plot showing *t*-test results at I_1570_/I_998 cm-1_ for cancer (OC) and H, respectively. Each scatter represents one SERS spectrum. (b) Hierarchical clustering heatmap. (c) Heatmaps of mean intensity ratios (I_1070_/I_998 cm-1_ and I_1570_/I_998 cm-1_) for 20 OC patients and 20 healthy individuals, with each strip representing one individual. (d) The *t*-SNE scatter plot of healthy individuals (H) and cancer (OC).


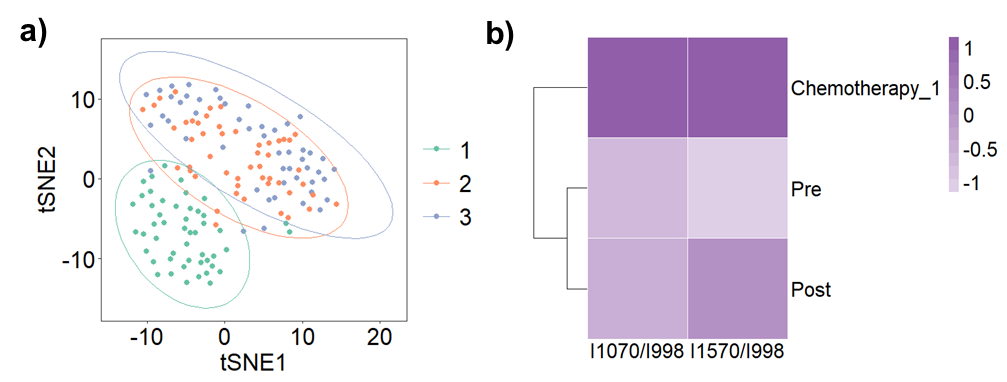


Figure S12. (a) The *t*-SNE scatter plots and (b) clustered heat maps (I_1070_/I_998_ _cm-1_ and I_1570_/I_998_ _cm-1_) during preoperative (1, Pre), postoperative (2, Post), and first chemotherapy (3, Che-I) of patient 1 (P1), respectively.


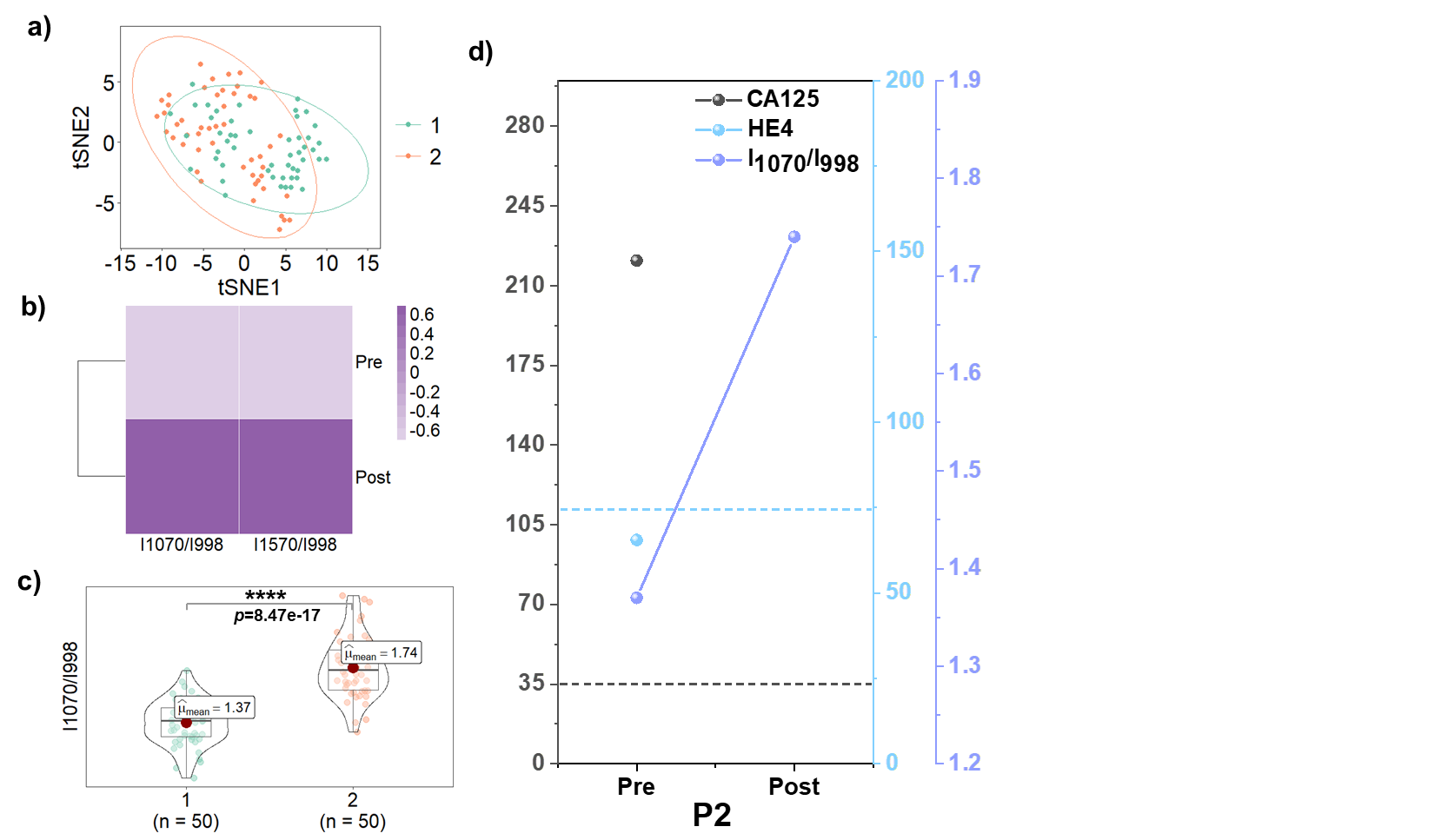


Figure S13. (a) The *t*-SNE scatter plots, (b) clustered heat maps (I_1070_/I_998_ _cm-1_ and I_1570_/I_998_ _cm-1_), and (c) violin scatter plots with *t*-test (I_1070_/I_998_ _cm-1_) during preoperative (1, Pre) and postoperative (2, Post) of patient 2 (P2), respectively. ****P < 0.0001, ***P < 0.001, **P < 0.01, *P < 0.05. (d) Line plots of clinical assay values for biomarkers CA125 (U/mL), HE4 (pmol/L) values from clinical tests at different treatment stages for P2, along with the SA (I_1070_/I_998_ _cm-1_) values. The dotted lines of different colors correspond to the reference values of the corresponding markers.


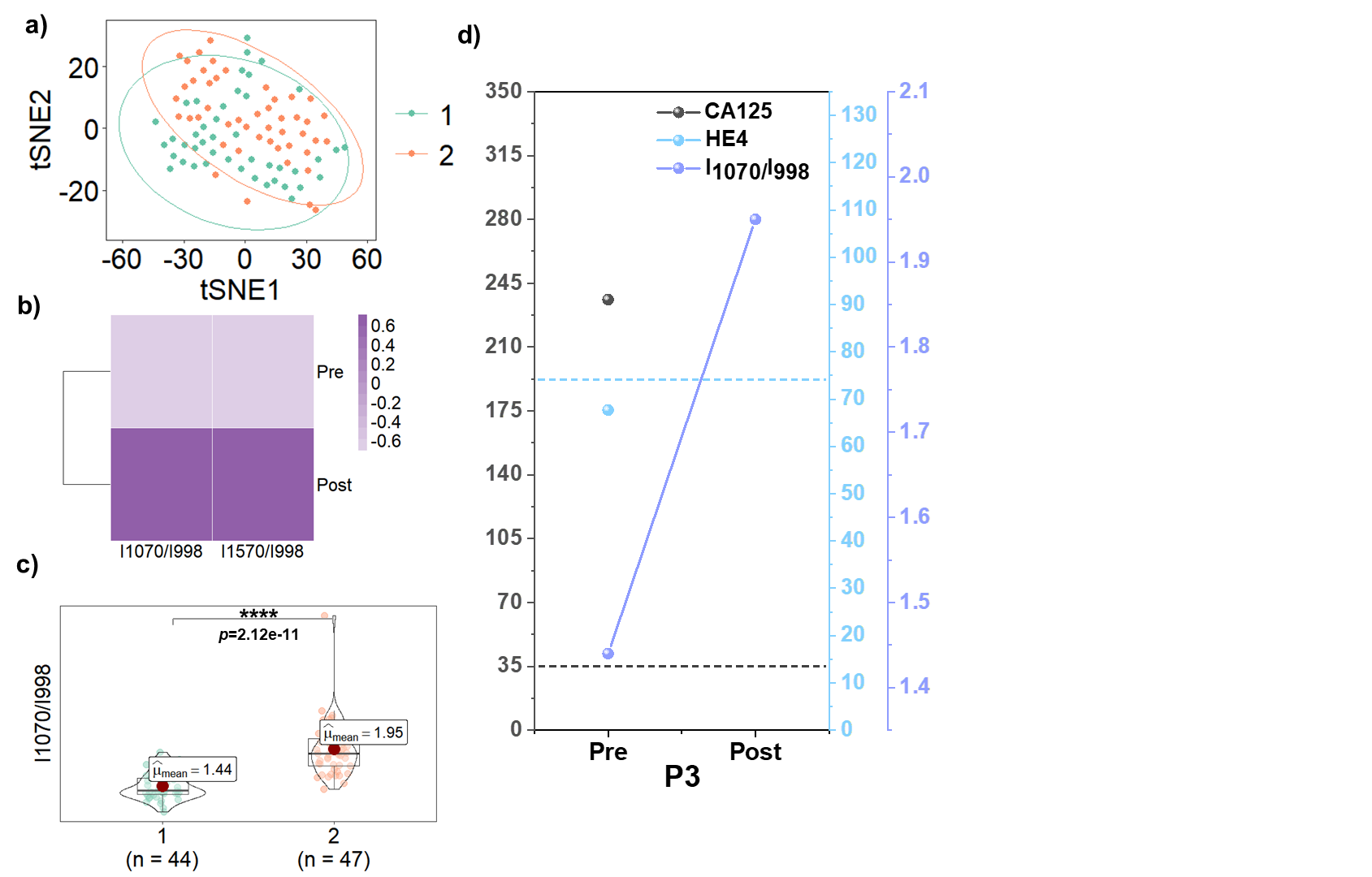


Figure S14. (a) The *t*-SNE scatter plots, (b) clustered heat maps (I_1070_/I_998_ _cm-1_ and I_1570_/I_998_ _cm-1_), and (c) violin scatter plots with *t*-test (I_1070_/I_998_ _cm-1_) during preoperative (1, Pre) and postoperative (2, Post) of patient 3 (P3), respectively. ****P < 0.0001, ***P < 0.001, **P < 0.01, *P < 0.05. (d) Line plots of clinical assay values for biomarkers CA125 (U/mL), HE4 (pmol/L) values from clinical tests at different treatment stages for P3, along with the SA (I_1070_/I_998_ _cm-1_) values. The dotted lines of different colors correspond to the reference values of the corresponding markers.


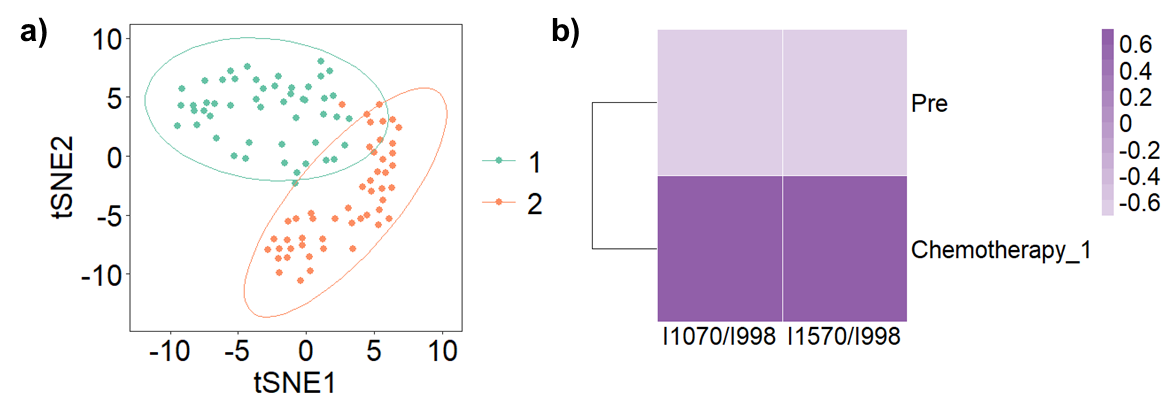


Figure S15. (a) The *t*-SNE scatter plots and (b) clustered heat maps (I_1070_/I_998_ _cm-1_ and I_1570_/I_998_ _cm-1_) during preoperative (1, Pre), and first chemotherapy (2, Che-I) of patient 4 (P4), respectively.


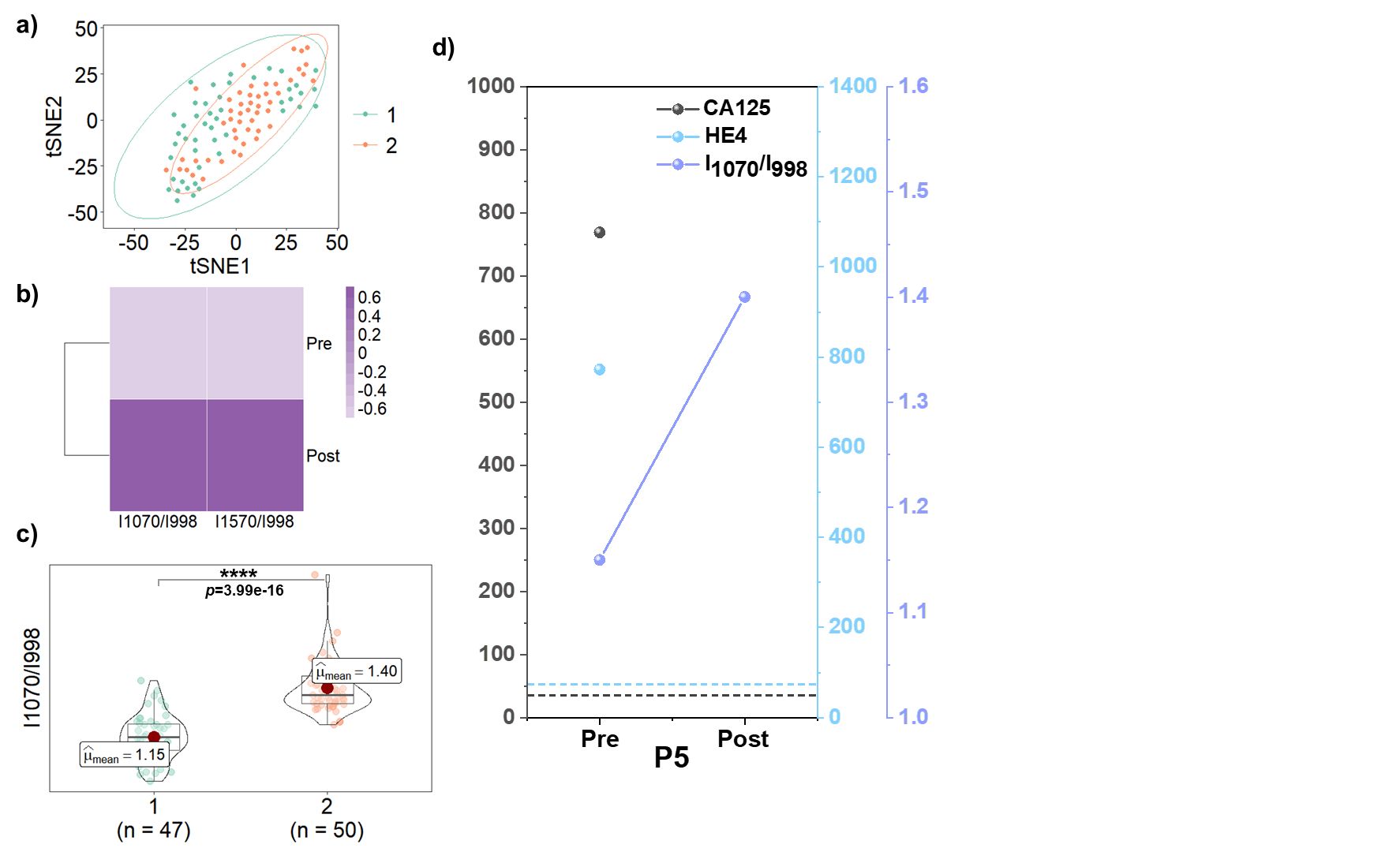


Figure S16. (a) The *t*-SNE scatter plots, (b) clustered heat maps (I_1070_/I_998_ _cm-1_ and I_1570_/I_998_ _cm-1_), and (c) violin scatter plots with *t*-test (I_1070_/I_998_ _cm-1_) during preoperative (1, Pre) and postoperative (2, Post) of patient 5 (P5), respectively. ****P < 0.0001, ***P < 0.001, **P < 0.01, *P < 0.05. (d) Line plots of clinical assay values for biomarkers CA125 (U/mL), HE4 (pmol/L) values from clinical tests at different treatment stages for P5, along with the SA (I_1070_/I_998_ _cm-1_) values. The dotted lines of different colors correspond to the reference values of the corresponding markers.


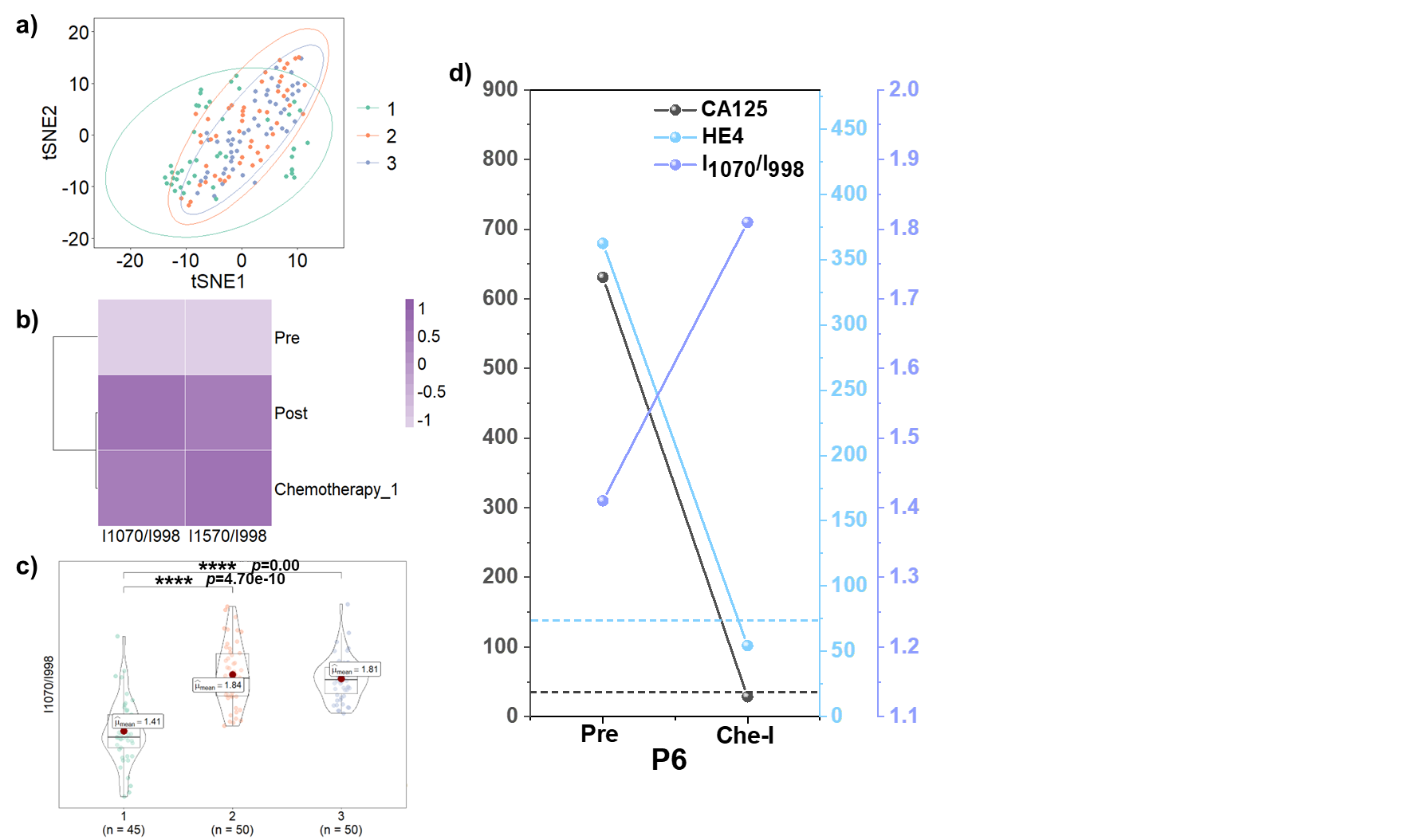


Figure S17. (a) The *t*-SNE scatter plots, (b) clustered heat maps (I_1070_/I_998_ _cm-1_ and I_1570_/I_998_ _cm-1_), and (c) violin scatter plots with *t*-test (I_1070_/I_998_ _cm-1_) during preoperative (1, Pre), postoperative (2, Post), and first chemotherapy (3, Che-I) of patient 6 (P6), respectively. ****P < 0.0001, ***P < 0.001, **P < 0.01, *P < 0.05. (d) Line plots of clinical assay values for biomarkers CA125 (U/mL), HE4 (pmol/L) values from clinical tests at different treatment stages for P6, along with the SA (I_1070_/I_998_ _cm-1_) values. The dotted lines of different colors correspond to the reference values of the corresponding markers.


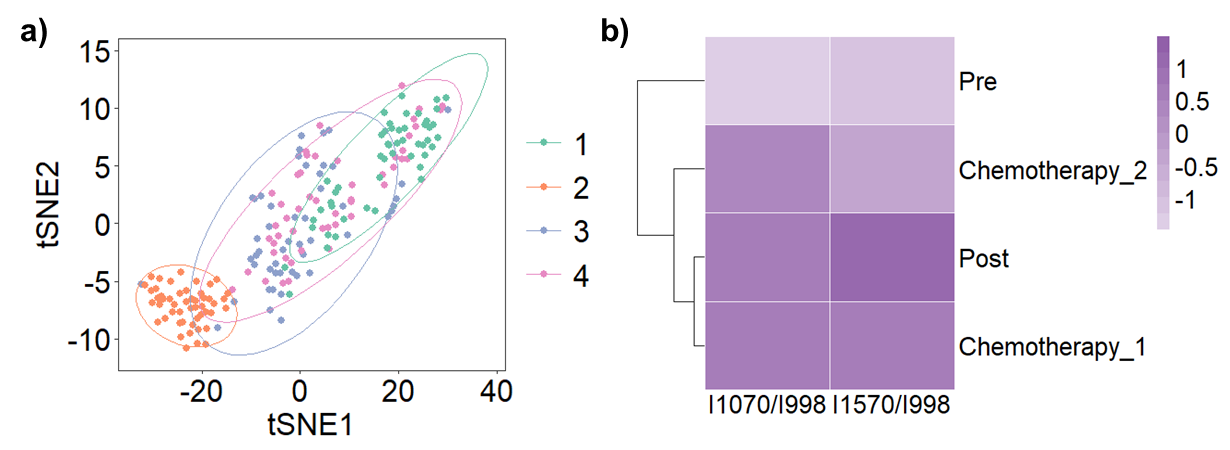


Figure S18. (a) The *t*-SNE scatter plots and (b) clustered heat maps (I_1070_/I_998_ _cm-1_ and I_1570_/I_998_ _cm-1_) during preoperative (1, Pre), postoperative (2, Post), first chemotherapy (3, Che-I), and second chemotherapy (4, Che-II) of patient 7 (P7), respectively.


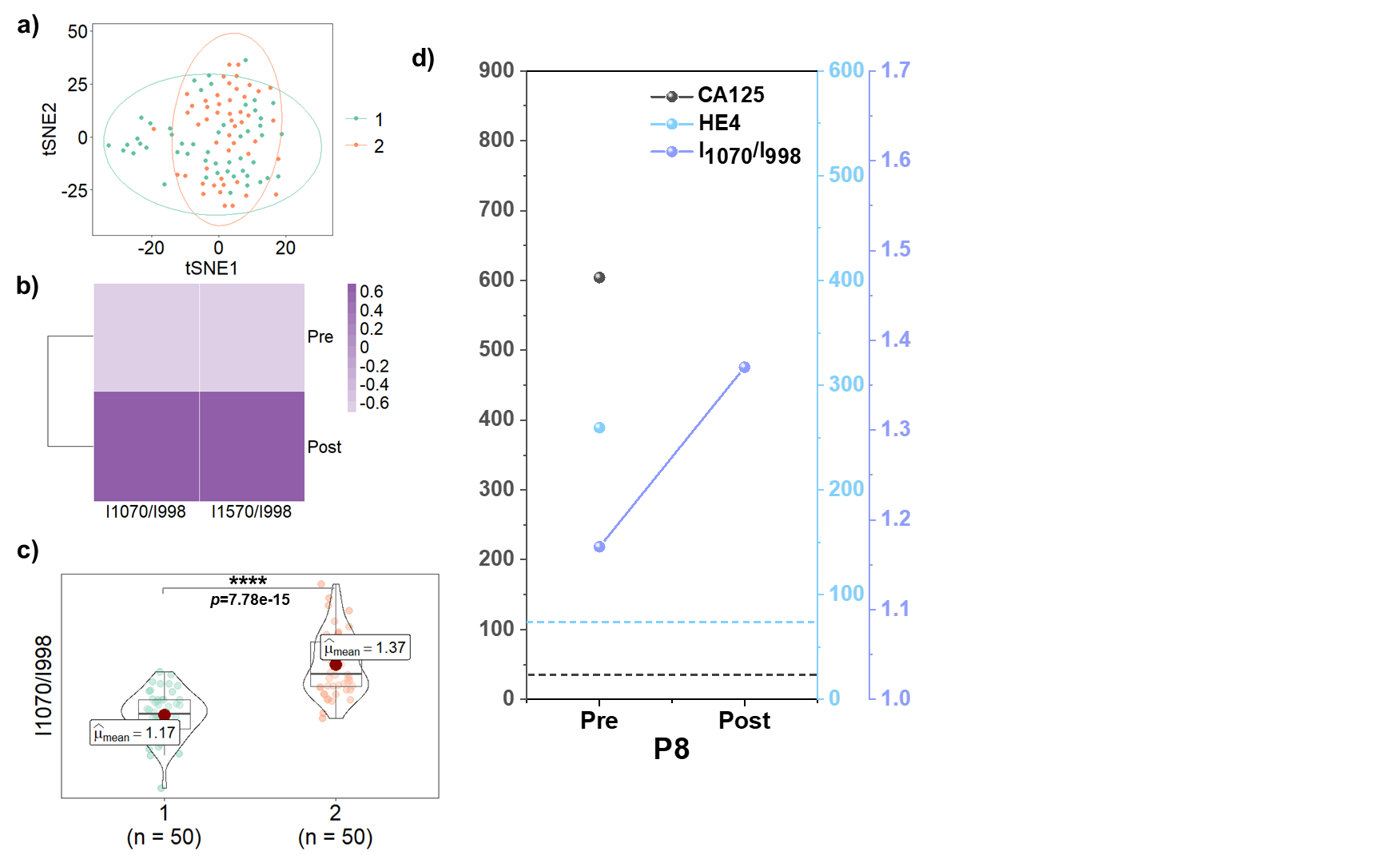


Figure S19. (a) The *t*-SNE scatter plots, (b) clustered heat maps (I_1070_/I_998_ _cm-1_ and I_1570_/I_998_ _cm-1_), and (c) violin scatter plots with *t*-test (I_1070_/I_998_ _cm-1_) during preoperative (1, Pre) and postoperative (2, Post) of patient 8 (P8), respectively. ****P < 0.0001, ***P < 0.001, **P < 0.01, *P < 0.05. (d) Line plots of clinical assay values for biomarkers CA125 (U/mL), HE4 (pmol/L) values from clinical tests at different treatment stages for P8, along with the SA (I_1070_/I_998_ _cm-1_) values. The dotted lines of different colors correspond to the reference values of the corresponding markers.


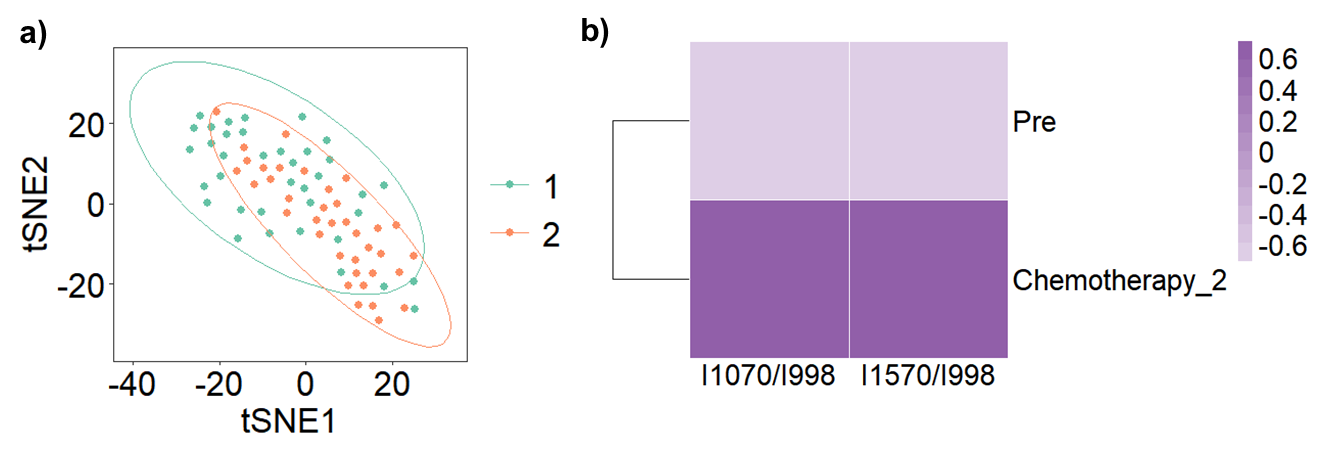


Figure S20. (a) The *t*-SNE scatter plots and (b) clustered heat maps (I_1070_/I_998_ _cm-1_ and I_1570_/I_998_ _cm-1_) during preoperative (1, Pre) and second chemotherapy (2, Che-II) of patient 9 (P9), respectively.

1. **Tables**

Table S1. The detailed band assignments of calculated Raman spectra, experimental Raman spectra, and SERS spectra for 4-MPBA.^2^

| Calculated  (cm^-1^) | Experimental  (cm^-1^) | SERS  (cm^-1^) | Modes  assignment |
| --- | --- | --- | --- |
| 648 | 631 | － | β_CH_ |
| － | 723 | 690 | β_CCC_ + ν_CS_ |
| 765 | 802 | － | γ_CH+_γ_CCC_ |
| 936-971 | 905 | 923 | β_CHS_ |
| 1014 | 1003,1024 | 998,1020 | β_CCC+_β_CH_ |
| 1107 | 1091-1106 | 1070 | β_CCC_ + ν_CS_+β_OH_ |
| － | 1183 | 1183 | β_CH_+β_BOH_ |
| 1374 | 1308-1367 | － | ν_BO_ |
| － | － | 1471 | ν_CC_ |
| 1646 | 1592 | 1570 | ν_CC_ |
| 2695 | 2564 | － | ν_SH_ |

Table S2. The clinically measured CA125 and HE4 values for each OC patient during different treatment periods.

| Patient Number | Period of  treatment | CA125 (U/mL)  (Ref. 0.00-35.00) | HE4 (pmol/L)  (Ref. 0.00-74.30) |
| --- | --- | --- | --- |
| P1 | Pre | 4120↑ | 692.3↑ |
|  | Post | － | － |
|  | Chemotherapy I | － | － |
| P2 | Pre | 221↑ | 65.53 |
|  | Post | － | － |
| P3 | Pre | 236↑ | 67.68 |
|  | Post | － | － |
| P4 | Pre | 3530↑ | 700.7↑ |
|  | Chemotherapy I | 178↑ | 101.9↑ |
| P5 | Pre | 769↑ | 772.3↑ |
|  | Post | － | － |
| P6 | Pre | 631↑ | 362.4↑ |
|  | Post | － | － |
|  | Chemotherapy I | 28.5 | 54.37 |
| P7 | Pre | 358↑ | 247.9↑ |
|  | Post | － | － |
|  | Chemotherapy I | 109↑ | 64.39 |
|  | Chemotherapy II | 22.3 | 53.81 |
| P8 | Pre | 604↑ | 259.3↑ |
|  | Post |  |  |
| P9 | Pre  Chemotherapy II | 251↑  270↑ | 430.1↑  71.01 |
| P10 | Pre | 252↑ | 53.71 |
|  | Post | 118↑ | 58.16 |
|  | Chemotherapy I | － | － |
|  | Chemotherapy II | － | － |

Table S3. The basic information for OC Patients.

| Patient Number | Age | Pathological types | FIGO stage |
| --- | --- | --- | --- |
| P1 | 64 | High-grade serous carcinoma | IVB |
| P2 | 52 | High-grade serous carcinoma | IC2 |
| P3 | 34 | Clear cell carcinomas | IVB |
| P4 | 58 | High-grade serous carcinoma | IIIC |
| P5 | 58 | High-grade serous carcinoma | IVB |
| P6 | 53 | High-grade serous carcinoma | IV |
| P7 | 58 | High-grade serous carcinoma | IVB |
| P8 | 73 | High-grade serous carcinoma | IIIC |
| P9 | 56 | High-grade serous carcinoma | IVB |
| P10 | 60 | High-grade serous carcinoma | IIIA1 |
| P11 | 55 | Low-grade serous carcinoma | IIIA |
| P12 | 63 | High-grade serous carcinoma | IIIB |
| P13 | 53 | High-grade serous carcinoma | IV |
| P14 | 53 | High-grade serous carcinoma | IIB |
| P15 | 48 | High-grade serous carcinoma | IIIC |
| P16 | 60 | High-grade serous carcinoma | IVB |
| P17 | 47 | High-grade serous carcinoma | IVB |
| P18 | 43 | High-grade serous carcinoma | IIIC |
| P19 | 65 | Clear cell carcinomas | IIIC |
| P20 | 52 | High-grade serous carcinoma | IIIC |

Table S4. Clinical tumor assessment for ovarian cancer patients.

| Patient Number | CA125 after the third chemotherapy  (Ref. 0.00-35.00) | HE4 after the third chemotherapy  (Ref. 0.00-74.30) | CT of the abdomen and lungs | Follow-up results | Tumor Assessment |
| --- | --- | --- | --- | --- | --- |
| P1 | 52.80 U/mL | 76.64 pmol/L | － | － | － |
| P2 | 8.5 U/mL | 38.4pmol/L | No lesions | － | CR |
| P3 | － | － | － | － | － |
| P4 | 114.00 U/mL | 84.91 pmol/L | Peritoneal, iliac vessels, and perisplenic metastases | － | PR |
| P5 | － | － | － | Normal | CR |
| P6 | 7.39 U/mL | 68.06 pmol/L | No lesions | － | CR |
| P7 | 11.70 U/mL | 47.30 pmol/L | No lesions | － | CR |
| P8 | － | － | － | Normal | CR |
| P9 | 54.30 U/mL | 54.1 pmol/L | No lesions | － | CR |

* ‘－’ indicates that the patient did not undergo examination at our hospital due to personal reasons.

1. **References**
2. Lee, P. C.; Meisel, D. Adsorption and surface-enhanced Raman of dyes on silver and gold sols. *J. Phys. Chem.* **1982,** *86* (17), 3391-3395.
3. Liang, L. J.; Shen, Y. T.; Zhang. J.; Xu, S. P.; Xu, W. Q.; Liang, C. Y.; Han, B. *Anal. Chim. Acta*. **2018,** 1033, 148-155.
